# C-terminal FGF-23 production coupling with aldosterone via FAM20C and predicting cardiovascular events in primary aldosteronism

**DOI:** 10.1172/jci.insight.166461

**Published:** 2025-02-24

**Authors:** Vin-Cent Wu, Kang-Yung Peng, Tsu-I Chen, Chiao-Yin Sun, Hung-Wei Liao, Chieh-Kai Chan, Yen-Hung Lin, Hung-Hsiang Liou, Jeff S. Chueh

**Affiliations:** 1 Department of Internal Medicine and; 2 Department of Urology, National Taiwan University Hospital, Taipei, Taiwan.; 3 Division of Nephrology, Department of Internal Medicine, Chang Gung Memorial Hospital, Keelung, Taiwan.; 4 Department of Internal Medicine, Wan-Fang Hospital, Taipei, Taiwan.; 5 Division of Nephrology, Department of Internal Medicine, National Taiwan University Hospital, Hsinchu City, Taiwan.; 6 Division of Nephrology, Department of Internal Medicine, Hsin-Jen Hospital, New Taipei City, Taiwan.

**Keywords:** Cardiology, Endocrinology, Cardiovascular disease, Hypertension

## Abstract

This study examined the involvement of fibroblast growth factor-23 (FGF-23) in primary aldosteronism (PA), a condition characterized by elevated aldosterone levels and hypertension. We recruited patients with unilateral PA (uPA) and observed increased levels of C-terminal FGF-23 (cFGF-23) and C-terminal to intact FGF-23 (iFGF-23) in patients with uPA compared with essential hypertension control participants. Elevated preoperative cFGF-23 levels were associated with adverse outcomes, including mortality and cardiovascular or kidney events. Plasma cFGF-23 levels demonstrated a nonlinear rise with aldosterone, but iFGF-23 levels were not correlated with plasma aldosterone concentration. Higher cFGF-23 levels independently predicted hypertension remission after adrenalectomy for patients with uPA. Patients with uPA, who exhibited elevated cFGF-23 levels, had decreased levels after adrenalectomy. In cell cultures, aldosterone enhanced cleavage of iFGF-23, leading to increased levels of cFGF-23 fragments, an effect mitigated by silencing of family with sequence similarity 20, member C (FAM20C). However, the enhancement of cFGF-23 levels remained unaffected by the furin inhibitor. The study suggests that aldosterone influences FGF-23 phosphorylation by interacting with FAM20C, with docking experiments indicating aldosterone’s binding to FAM20C. This work highlights that patients with uPA with elevated cFGF-23 levels are associated with cardiovascular risks, and adrenalectomy reduces cFGF-23. Aldosterone likely promotes cFGF-23 production through FAM20C-mediated phosphorylation of iFGF-23.

## Abstract 2

Aldosterone coupling FGF-23 via FAM20C in PA

## Graphical abstract

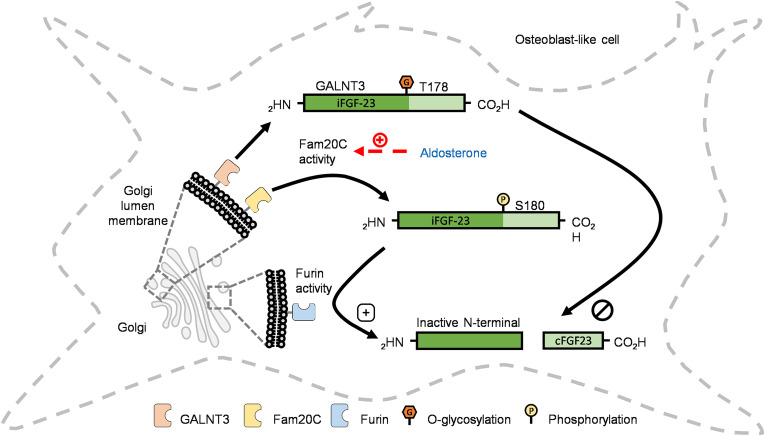

## Introduction

Fibroblast growth factor-23 (FGF-23) was initially recognized for its phosphaturic function in rare hypophosphatemia disorders (1), with elevated levels associated with various adverse cardiovascular outcomes, such as upregulation of the renin-aldosterone-angiotensin system (RAAS) (2, 3), volume expansion (4), endothelial dysfunction (5), and left ventricular hypertrophy (6). Moreover, higher FGF-23 levels independently correlate with an increased risk of incident hypertension and all-cause mortality over time (7–9).

Aldosterone has been found to upregulate FGF-23 transcripts in osteoblastic cells, a process reversed by mineralocorticoid receptor antagonist (MRA) treatment in animal models (10). FGF-23, in turn, interacts with phosphorus to form a negative feedback loop, where high phosphorus levels stimulate FGF-23 production, which acts on the kidney to lower phosphorus levels (11). Additionally, aldosterone-mediated upregulation has been linked to higher FGF-23 concentrations in chlorothiazide-sensitive Na+:Cl– cotransporter–knockout mice (12). Furthermore, FGF-23 can activate the local RAAS and induce cardiac fibrosis, a process ameliorated by spironolactone (an MRA) (13), suggesting a potential positive interactive loop between aldosterone and FGF-23.

FGF-23 activity is regulated not only by its production but also by posttranslational modification, involving the secretory process of FGF-23; dynamic regulation of family with sequence similarity 20, member C (FAM20C); and/or furin proteolysis. Differential cleavage by furin regulates the ratio of intact FGF-23 (iFGF-23) to C-terminal FGF-23 (cFGF-23) (14), where intracellular cleavage depends on the competition between glycosylation via polypeptide *N*-acetylgalactosaminyltransferase 3 (GALNT3), inhibiting cFGF-23 production, and phosphorylation via FAM20C, promoting cFGF-23 production (15, 16).

Primary aldosteronism (PA) prevalence in patients with resistant hypertension ranges from 5% to 13% (17), characterized by autonomous aldosterone overproduction with suppressed plasma renin activity (PRA). Patients with PA face a significantly higher cardiovascular risk compared with age-, sex-, and blood pressure–matched patients with essential hypertension (EH). This study aims to elucidate the roles and effects of FGF-23 and its protein cleavage in patients with unilateral PA (uPA).

## Results

### Clinical characteristics of the patients and controls.

**Figure 1:**
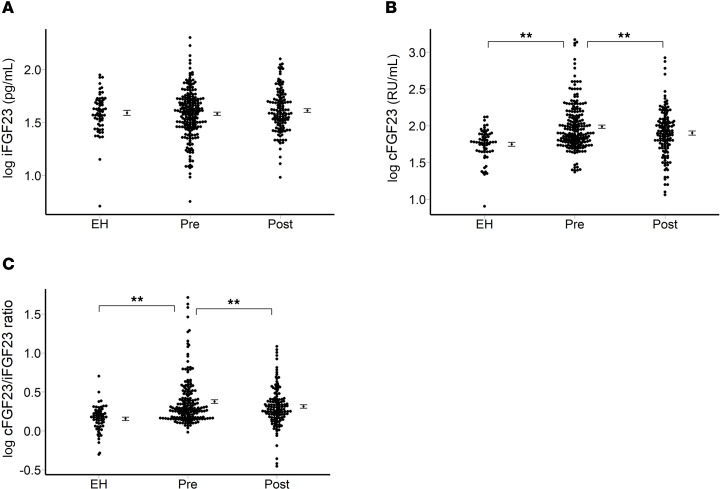
FGF-23 levels assessed in patients both before and after adrenalectomy and compared with matched patients with EH. Plasma levels of log-transformed (**A**) iFGF-23 and (**B**) cFGF-23 from 121 patients with uPA. (**C**) The cFGF-23/iFGF-23 ratio (*n* = 69). Statistical analysis was conducted using 1-way ANOVA with Bonferroni’s post hoc correction for multiple comparisons (*P < 0.05 or **P < 0.01 vs. preoperative). Graphical data are shown as mean ± SEM. cFGF-23, C-terminal fibroblast growth factor-23; EH, essential hypertension; iFGF-23, intact fibroblast growth factor-23.

In this study, a total of 121 patients with uPA (with a mean age of 51.0 years; 61 women, accounting for 54.5%) were included, alongside 69 EH control participants matched for age, sex, estimated glomerular filtration rate (eGFR), and body weight. The log-transformed cFGF-23 level was significantly elevated in patients with uPA compared with EH controls (1.98 ± 0.33 vs. 1.74 ± 0.21; *P* < 0.01) and notably decreased after adrenalectomy (1.89 ± 0.32, *P* < 0.01), while the log iFGF-23 level remained almost unchanged (Figure 1, A and B). Additionally, the ratio of cFGF-23 to intact FGF-23 (c/i FGF-23 ratio) was higher in patients with uPA compared with EH controls (log-transformed 0.37 ± 0.30 vs. 0.16 ± 0.17; *P* < 0.01). Following adrenalectomy, the c/i FGF-23 ratio significantly decreased (0.30 ± 0.25; *P* < 0.01) (Figure 1C). Furthermore, patients with uPA exhibited higher intact parathyroid hormone (iPTH) levels (*P* < 0.001) and lower total calcium levels (*P* < 0.001) compared with EH controls (Table 1).

### Association between aldosterone and cFGF-23 levels.

**Figure 2:**
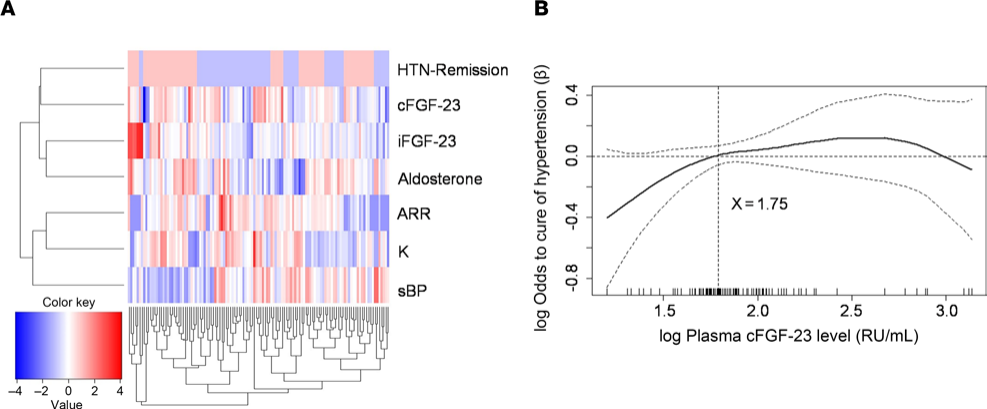
Biomarkers and clinical success in patients with uPA after adrenalectomy. (**A**) Biomarkers related to complete clinical success (*n* = 121), analyzed by unsupervised clustering to determine their relationship to complete clinical success. Full-length view of the cluster diagram has cases orientated along the *x* axis and biomarkers orientated along the *y* axis. A GAM picture depicts (**B**) the probability of hypertension remission constructed with plasma cFGF-23 level that has an average of 0 over the range of the data, i.e., log [cFGF-23] = 1.75 (*n* = 121). The dashed lines indicate approximated pointwise 95% CI. Data were compared by Fisher’s exact test. ARR, aldosterone-to-renin ratio; CI, confidence interval; GAM, generalized additive model; HTN, hypertension; K, potassium; RU, relative units; sBP, systolic blood pressure.

In a multivariate-adjusted generalized additive model (GAM) plot, plasma cFGF-23 levels exhibited a nonlinear increase relative to plasma aldosterone concentration (PAC). Specifically, when the log[PAC] level exceeded 1.98, cFGF-23 levels markedly rose in patients with PA (refer to Supplemental Figure 1; https://doi.org/10.1172/jci.insight.166461DS1; and Figure 2A). Conversely, plasma iFGF-23 levels showed no significant association with PAC (Supplemental Figure 1 and Figure 2B; supplemental material available online with this article), indicating independent level and cleavage mechanisms for iFGF-23, thus ruling out collinearity between cFGF-23 and iFGF-23 levels.

Subsequently, patients with uPA were stratified into high or low (preoperative) cFGF-23 level subgroups based on a log [cFGF-23] cutoff value of 1.98. Notably, eGFR, aldosterone, renin, 25 OH vitamin D, 1,25(OH)_2_ vitamin D, calcium, and iPTH levels were comparable between these 2 subgroups (Table 2).

### Baseline cFGF-23 levels are associated with cardiovascular composite outcomes.

Following a mean follow-up period of 4.7 ± 2.9 years, among the patients with uPA, 3 individuals (2.5%) succumbed, 9 (7.4%) experienced stroke, and 5 (4.1%) experienced coronary artery diseases. In the Cox proportional hazard model, a preoperative log [plasma cFGF-23] > 1.98 (yes) (hazard ratio [HR] = 4.2, 95% confidence interval [CI] = 1.71–10.2; *P* = 0.038), systolic blood pressure (HR = 1.06, 95% CI = 1.03–0.09, *P* < 0.001), and duration of hypertension (HR = 1.10, 95% CI = 1.06–1.13, *P* = 0.036) emerged as statistically significant risk factors associated with cardiovascular composite outcomes (Supplemental Table 2). The relative risk for all-cause mortality, cardiovascular events, and kidney events was 2.63, with an *E*-value for the point estimates of 4.71. This analysis suggested a limited potential for substantial unmeasured confounding.

### Hypertension remission after adrenalectomy.

Among the patients with uPA, 62 individuals (51.2%) experienced hypertension remission based on the Primary Aldosteronism Surgical Outcome (PASO) definition. Plasma levels of cFGF-23 (AUC = 0.844), iFGF-23 (AUC = 0.767), c/i FGF-23 ratio (AUC = 0.724), and PAC (AUC = 0.617) at the time of PA confirmation were predictive of hypertension remission following adrenalectomy, with cFGF-23 level demonstrating superior predictive ability.

Given the possibility of collinearity among certain biomarkers, an unsupervised cluster analysis was conducted to assess their relationships with hypertension remission. Preoperative plasma cFGF-23 level was found to be closely associated with the likelihood of hypertension remission, which may explain its robust predictive performance for clinical remission.

A GAM plot illustrated a positive nonlinear correlation between elevated preoperative plasma cFGF-23 levels and the log odds of hypertension remission 1 year after adrenalectomy. After adjusting for nonlinear effects of various factors such as age, sex, aldosterone, blood pressure, eGFR, and iPTH, preoperative log [cFGF-23] level with a cutoff value ≥ 1.75 was found to independently predict hypertension remission.

Interestingly, patients with uPA in the high cFGF-23 subgroup, with log-transformed cFGF-23 level ≥ 1.75, exhibited a greater likelihood of hypertension remission following adrenalectomy compared with those in the low cFGF-23 subgroup (*P* < 0.001). However, there was no significant difference in biochemical cure rates between the 2 subgroups (*P* = 0.238).

Logistic regression analysis, adjusted for confounding factors, revealed that patients with lower systolic blood pressure (odds ratio [OR] = 0.96, 95% CI = 0.93–0.98, *P* = 0.001), absence of diabetes (OR of having diabetes = 0.27, 95% CI = 0.08–0.91, *P* = 0.035), and higher preoperative baseline cFGF-23 (OR = 3.8, 95% CI = 1.5–9.6, *P* = 0.004) had significantly improved odds of achieving hypertension remission after adrenalectomy.

### cFGF-23 levels increase in bone cells treated with aldosterone.

**Figure 3:**
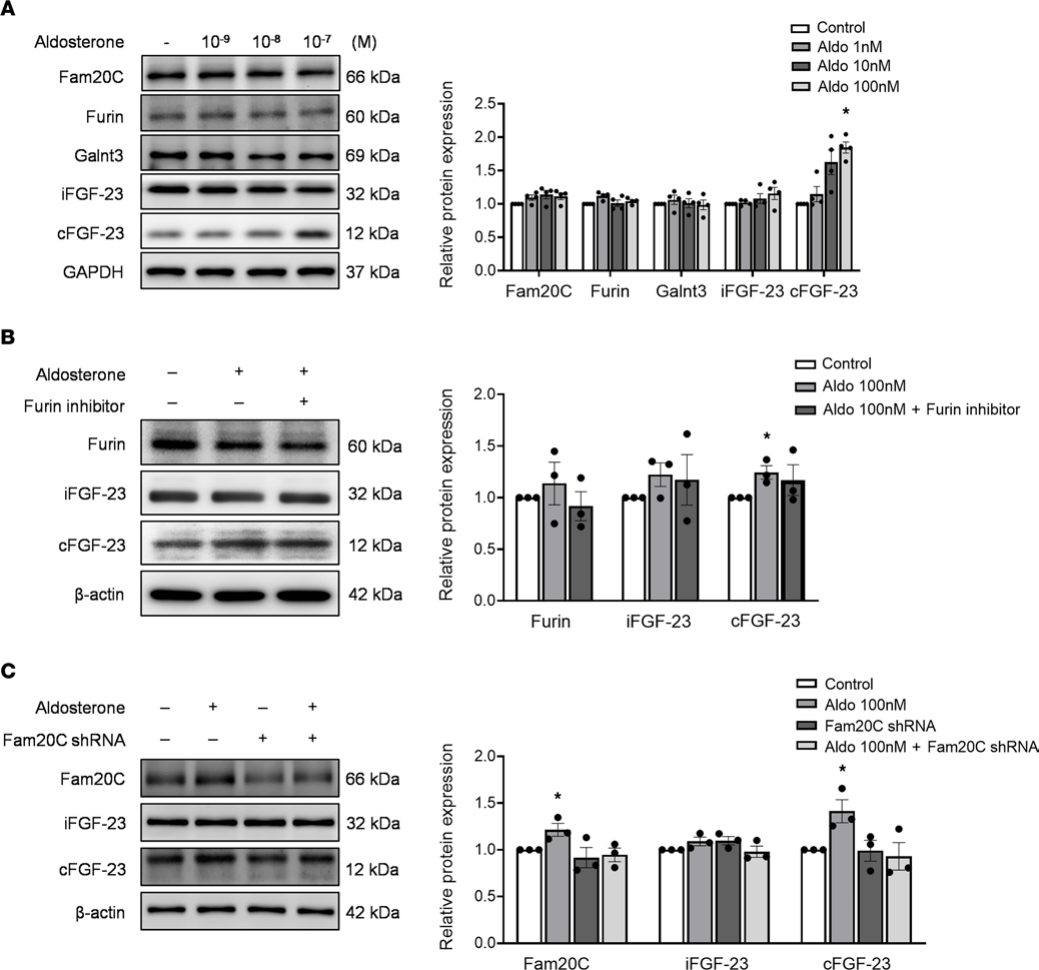
The level of furin, Galnt3, Fam20C, iFGF-23, and cFGF-23 in UMR-106 cells after aldosterone treatment. (**A**) The dose-dependent effect of aldosterone on the upregulation of cFGF-23, *n* = 4/group. (**B**) The upregulation of cFGF-23 after aldosterone treatment remains unaffected by the furin inhibitor, *n* = 3/group. (**C**) Silencing Fam20C (shRNA) effectively restored the upregulated cleavage of cFGF-23, *n* = 3/group. Statistical analysis was conducted using 1-way ANOVA with Bonferroni’s post hoc correction for multiple comparisons (*P < 0.05, vs. control group). The graph data are presented as mean ± SEM.

Our findings revealed a rapid surge in cFGF-23 after aldosterone treatment, while levels of iFGF-23 remained relatively unchanged. This observation strongly suggests that the heightened production of cFGF-23 is intricately linked to posttranslational modifications of iFGF-23. Such modifications likely serve to prevent a concurrent increase in the expression of iFGF-23. The consistent findings observed in another osteocyte cell line, IDG-SW3, mirrored those seen in UMR-106 cells (Supplemental Figure 3). Western blot analysis revealed an increase in cFGF-23 levels following aldosterone treatment ranging from 10^–9^ to 10^–7^ M (Figure 3A). Notably, this effect persisted even after the addition of a furin inhibitor at 10^–6^ M (Figure 3B). However, the expression of iFGF-23 remained unchanged after coculture with aldosterone. It is worth mentioning that furin levels remained unaffected by aldosterone treatment. Moreover, an assay measuring furin-like enzyme activity in cell lysates showed no significant change in total furin-like enzyme activity following different doses of aldosterone treatment, compared to controls, or furin inhibitor treatment (Supplemental Figure 4). Following aldosterone treatment, there was a notable increase in the protein expression of FAM20C (1.21-fold ± 0.1-fold, *P* = 0.036). To explore the impact of aldosterone on iFGF-23 phosphorylation via Fam20C, shRNA targeting Fam20C was introduced into the UMR-106 culture. The elevated level of cFGF-23 (1.41-fold ± 0.17-fold, *P* = 0.028) observed after aldosterone infusion was mitigated upon cotreatment with Fam20C shRNA (*P* = 0.659) (0.5 μg plasmid for 16 hours) (Figure 3C). These findings support the active involvement of FAM20C in the proteolytic processing of iFGF-23 following aldosterone stimulation.

### Interaction between FAM20C and FGF-23 after aldosterone treatment.

**Figure 4:**
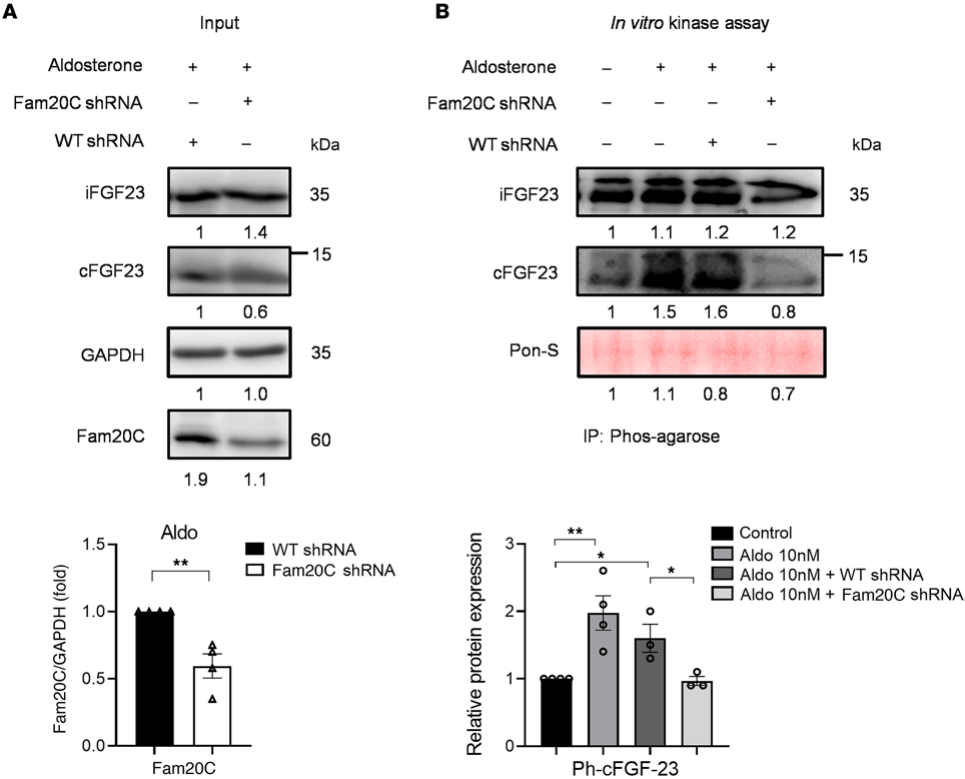
Aldosterone exerts a stimulatory effect on FGF-23 by modulating Fam20C activity. (**A**) Aldosterone led to increased cFGF-23 levels compared with shFam20C-transfected cells in UMR-106 cells. (**B**) After phos-agarose immunoprecipitation, aldosterone promotes interactions between FGF-23 and Fam20C-WT, accompanied by the expected phosphorylation. *n* = 3/group. Ponceau S is a dye used to stain as internal control. *P < 0.05, **P < 0.01 by 1-way ANOVA with Bonferroni’s post hoc correction for multiple comparisons. Graph data are shown as mean ± SEM.

Upon transient transfections with shRNA Fam20C in UMR-106 cells, the interaction between Fam20C and FGF-23 in cell cultures was observed. The presence of aldosterone led to a noticeable increase in the cFGF-23 level compared with that of shFam20C-transfected cells (*P* < 0.01) (Figure 4A).

Subsequent to phos-agarose immunoprecipitation, aldosterone facilitates interactions between FGF-23 and Fam20C-WT. Aldosterone-induced augmentation of cFGF-23 levels was significantly attenuated in the presence of shFam20C (*P* < 0.05). This interaction was concomitant with the expected phosphorylation (Figure 4B).

### Aldosterone-infused mice and PTH inhibitor (cinacalcet) administration.

We assessed the impact of aldosterone on circulating cFGF-23 levels and its potential reversibility with a PTH inhibitor, cinacalcet. Specifically, we observed that aldosterone induces a proportional increase in FGF-23 cleavage. Consequently, while cFGF-23 levels exhibit a notable rise, iFGF-23 levels demonstrate a relative increase, yielding a c/i FGF-23 ratio approximating 1 in the animal model. Notably, the heightened transcription of iFGF-23 was restored by treatment with a PTH inhibitor, suggesting a potential role for PTH in modulating iFGF-23 production (Supplemental Figure 5).

### Docking interaction of aldosterone and FAM20.

**Figure 5:**
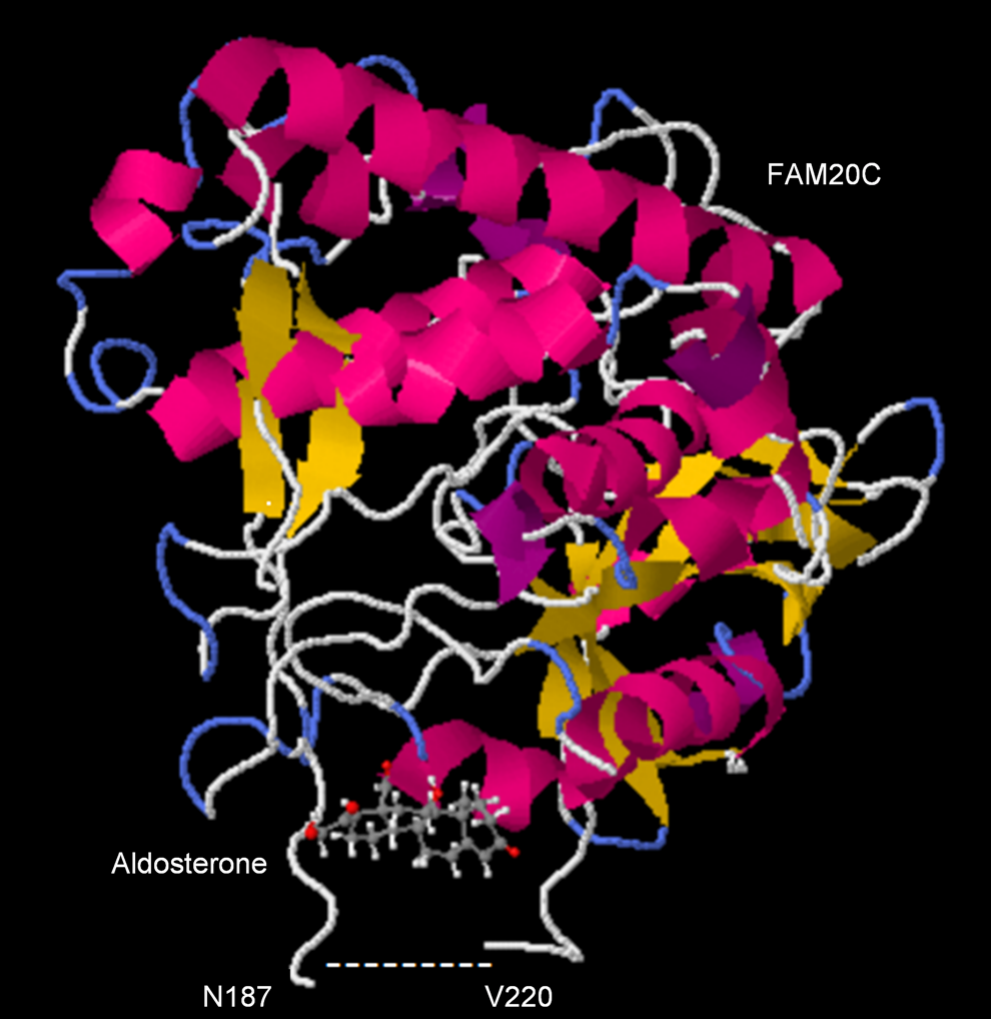
Aldosterone-induced modulation of FAM20C kinase activity in PA. Molecular docking results of FAM20C (PDB: 5YH3) and aldosterone. Hydrogen bonds are indicated by dashed lines. (Full fitness: –2,246.46 kcal/mol; estimated ΔG: –6.80 kcal/mol.) The blue line indicates hydrogen bonding, amino acids in green color indicate van der Waals interactions, and the white line represents a hydrogen bond.

Docking simulations revealed that aldosterone has the potential to bind to the putative drug binding site of FAM20C (PDB: 5YH3), with a full fitness score of –2,246.46 kcal/mol and an estimated change in Gibbs free energy (ΔG) of –6.80 kcal/mol. The plausible hormone binding pocket of FAM20C is situated opposite to the contact interface of FAM20C and binds with FAM20A. As depicted in Figure 5, aldosterone was positioned within the FGF-23 binding pocket, with its polar substituent situated at the inner region of the pocket, forming hydrogen bond interactions. These findings suggest that aldosterone could influence the kinase activity or FGF-23 binding capability of FAM20C through physical interactions.

## Discussion

In this study, we initially demonstrated that patients with uPA exhibited significantly elevated preoperative plasma cFGF-23 levels compared with EH controls, independent of kidney function and hyperparathyroidism. Interestingly, while higher preoperative cFGF-23 levels were associated with hypertension remission after adrenalectomy, they also predicted a heightened risk of long-term mortality or incident cardiovascular events. Our in vitro model provided further support for this hypothesis by indicating that the increased production of cFGF-23 resulted from enhanced posttranslational processing of iFGF-23, facilitated by aldosterone-induced upregulation of FAM20C activity and altered binding affinity. We elucidated how aldosterone influences FGF-23 phosphorylation by interacting with FAM20C. Moreover, structural docking and biochemical analyses shed light on the role of FAM20 kinases, revealing that aldosterone selectively binds and interferes with phosphorylated pro-kinases (Supplemental Figure 6).

### Aldosterone augments cFGF-23 levels in uPA.

Our findings reveal an augmented activity of FAM20C, promoting a phosphorylation modification that facilitates cleavage in osteoblast cell cultures following aldosterone exposure. This suggests that while iFGF-23 level remains unaltered in hyperaldosteronism, its cleavage and conversion into cFGF-23 are upregulated. We have demonstrated the potential influence of iPTH on FGF-23 production and provided animal studies to support the notion that aldosterone’s impact on FGF-23 may not primarily be mediated by PTH.

Furthermore, our results propose a potentially novel hormonal regulatory mechanism for the interaction between aldosterone and FGF-23 in patients with uPA. In this mechanism, aberrant stimulation of aldosterone could trigger cFGF-23 production and secretion from osteoblast cells. This could create a positive feedback loop, wherein elevated FGF-23 levels stimulate renin production, further augmenting aldosterone secretion (18). Additionally, FAM20C may posttranslationally phosphorylate iFGF-23, rendering it susceptible to proteolysis and resulting in increased secretion of cFGF-23 fragments (19). The stable level of iFGF-23 in our in vitro study suggests that the production of cFGF-23 is driven by an accelerated rate of phosphorylation modification by FAM20C in patients with hyperaldosteronism.

Aldosterone, being a steroid hormone, has been previously implicated in influencing kinase activity upon binding. Studies on protein kinase C have shown that steroid hormones selectively activate pro-kinase activity (20, 21). FAM20 proteins are novel kinases responsible for phosphorylating secreted proteins and proteoglycans. FAM20C is activated by forming an evolutionarily conserved homodimer or heterodimer with FAM20A (22). Our study uniquely demonstrates that aldosterone can disrupt the structure of Fam20C on the opposite binding surface of FAM20A. This underscores that the interaction between aldosterone and FGF-23, leading to the transition to cFGF-23, is intricately dependent on the catalytic capability of FAM20C. The S180 site is renowned for its pivotal role in the Fam20C-mediated actions on FGF-23 (15). Notably, our shFam20C interventions led to a discernible reduction in cFGF-23 cleavage and illustrated the interactions induced by aldosterone with Fam20C. It underscores that the interaction between aldosterone and iFGF-23, leading to the transition to cFGF-23, is inherently contingent upon the catalytic prowess of Fam20C.

The levels of phosphate and 1,25(OH)_2_ vitamin D were found to be comparable between the uPA and EH groups in our study, consistent with previous findings. These factors did not exert a significant influence on FGF-23 levels, particularly in patients with normal renal function. Experimental evidence suggests that in individuals with chronic kidney disease (CKD), elevated FGF-23 levels diminish calcitriol synthesis and contribute to the activation of the RAAS, potentially leading to increased renin production (23). However, our study exclusively included patients with uPA with normal kidney function, most of whom likely exhibited renal hyperfiltration (24) with suppressed renin levels. This outcome hints at a potential interplay between aldosterone and FGF-23.

Prior research has indicated that cFGF-23 levels rise before the onset of CKD-related hyperparathyroidism, suggesting its role as an early-stage CKD marker (25). Similarly, our patients with uPA displayed higher iPTH levels compared with patients with EH. It is known that iPTH can enhance plasma cFGF-23 levels but does not affect iFGF-23 levels (26, 27). Despite adjusting our model for plasma iPTH levels, calcium, phosphate, and kidney function, we observed a significant association between aldosterone and increased cFGF-23 levels. Notably, aldosterone-infused mice exhibited elevated plasma cFGF-23 levels, which remained unaffected by treatment with a PTH inhibitor. Therefore, our study reveals a finding regarding the pivotal and independent influence of hyperaldosteronism on increased cFGF-23 production.

### Preoperative cFGF-23 and outcomes of interest.

Although higher preoperative cFGF-23 levels were indicative of hypertension remission following adrenalectomy in patients with uPA, this did not mitigate the risk of long-term cardiovascular events or all-cause mortality after surgery. FGF-23, a protein reflecting physiological axes, has been shown to suppress the kidney expression of angiotensin-converting enzyme 2 (3), a negative regulator of the RAAS.

Elevated FGF-23 levels have also been linked to a higher risk of cardiovascular events or all-cause mortality in patients with cardiovascular disease (28). In patients with nondialysis CKD, cFGF-23 level was associated with risk of dialysis or mortality (29) and could improve prediction of risks for all-cause mortality and heart failure–related admission (9). Elevated cFGF-23 levels have been associated with a gradual increase in blood pressure and incident hypertension, even after adjustment for cardiovascular disease risk factors (7). High cFGF-23 levels are associated with inflammation (30), endothelial dysfunction (5), arterial wall stiffness (31), and factors that may increase peripheral resistance, all characteristic of early aldosterone-related vascular injury. Given these compelling data, the increase in cFGF-23 levels may indicate heightened FGF-23 synthesis or cleavage. Both processes can disrupt phosphate and vitamin D equilibrium, intensify RAAS activation, increase iron availability during acute inflammation, and contribute to cardiac remodeling (32–34). Notably, an elevated level of cFGF-23 is associated with prevalent anemia, changes in hemoglobin over time, and the development of anemia (35).

While the study findings indicate that elevated plasma cFGF-23 levels predict hypertension remission following adrenalectomy, there may be other contributing factors, such as the duration of hypertension prior to adrenalectomy, that also increase the risk of cardiovascular composite outcomes. The longer the effective treatment is delayed, the greater the potential for harm, as indicated by our Cox model. In clinical practice, patients with PA often experience delayed diagnosis and subsequent adrenalectomy, exacerbating these risks. This delay in addressing the condition can lead to adverse consequences that impact cardiovascular health and overall clinical outcomes.

### Study limitations.

While we aimed to minimize selection bias by including all consecutive patients with uPA with adequate renal function who underwent adrenalectomy during the study period, the observational nature of our identification of patients with uPA undergoing adrenalectomy remains susceptible to potential indication bias for the procedure. As FGF-23 is predominantly excreted in patients with normal kidney function (24), the plasma cFGF-23 levels in our patients with uPA, who had normal kidney function, were approximately 2-fold higher than those in the EH controls. However, even this 2-fold increase in FGF-23 could have detrimental effects. Due to the utilization of different units (pg/mL versus RU/mL) in the various detection kits for different forms of FGF-23, conversion equations to assess the numerical relationships among iFGF-23, cFGF-23, and total FGF-23 were not available in our study. Therefore, we were unable to evaluate the precise numerical associations among these forms of FGF-23.

### Perspectives.

To the best of our knowledge, no previous study has explored the interaction between the RAAS and FGF-23 or their potential roles in predicting treatment outcomes for patients with uPA following adrenalectomy. The RAAS stimulates various tubular sodium cotransporters, which are also targets of FGF-23 and its coreceptor, klotho (10). However, our study found that levels of klotho and vitamin D were comparable between our patients with uPA and EH. Further comprehension of the interplay among hypertension, aldosterone, and inflammation, considering potential undiscovered aspects of FGF-23 biology, is warranted. In this context, the regulation of FGF-23 by aldosterone and its implications for bone mineralization may represent another facet of the bone/kidney/endocrine axis, wherein FGF-23 coordinates both bone mineralization and renal phosphate handling. This may partially elucidate the heightened risk of bone fractures observed in PA (36). The complete composition of the cleavage machinery involved in iFGF-23 cleavage remains undefined, and the mechanisms underlying the upregulation or enhancement of iFGF-23 cleavage in patients with uPA are not yet understood. We propose a critical model wherein hyperaldosteronism contributes to elevated plasma cFGF-23 levels in patients with uPA, a phenomenon that may also be pertinent to secondary hyperaldosteronism, such as in congestive heart failure, liver failure, and many patients with CKD. Notably, increased aldosterone levels have been positively correlated with higher cFGF-23 levels in CKD across various species (37). Elevated FGF-23 levels could activate mineralocorticoid receptors, augment the effects of aldosterone, and exacerbate adverse effects on the kidneys and heart (38). Furthermore, our findings suggest a potential alternative pathway, independent of phosphate regulation, to reduce FGF-23 levels. This is particularly relevant given the significant morbidity and mortality associated with elevated plasma cFGF-23 levels. Additional investigations into their interactions and dose-response associations may unveil further intricate pathophysiological interplay.

### Conclusion.

This research presents the initial evidence indicating that elevated plasma cFGF-23 levels in patients with uPA are not contingent on kidney function, vitamin D levels, or hyperparathyroidism. Notably, patients with uPA displayed a positive nonlinear correlation between plasma cFGF-23 levels and PAC, whereas no such correlation was observed with iFGF-23. We propose that the heightened activity of FAM20C induced by aldosterone may serve as a pivotal mechanism in patients with uPA, leading to the elevation of plasma cFGF-23. This phenomenon is likely mediated through direct interactions between aldosterone and FAM20C, resulting in the augmentation of its cleavage. From a clinical standpoint, increased preoperative plasma cFGF-23 levels were positively correlated with hypertension remission after adrenalectomy in patients with unilateral PA. However, heightened cFGF-23 levels also indicated an elevated risk of composite outcomes, including mortality and cardiovascular events in patients with PA.

## Methods

### Sex as a biological variable.

In the human study, both male and female participants were included to ensure comprehensive representation of sex as a biological variable.

### Study population.

We investigated an inception cohort from the Taiwan Primary Aldosteronism Investigation (TAIPAI) group, utilizing a prospectively established PA database (39–41). Between August 2015 and January 2017, all patients with uPA with an eGFR exceeding 60 mL/min/1.73 m^2^ who underwent adrenalectomy were consecutively recruited and subsequently monitored for at least 1 year postoperatively. Follow-up continued for all participants until January 2019. The eGFR was determined using the Modification of Diet in Renal Disease equation (42). Diagnosis of PA was established through screening and confirmatory tests, including the saline infusion test or captopril test, followed by subtype identification.

The TAIPAI group enrolled patients from 2 medical centers, 3 affiliated hospitals, and 2 regional hospitals across various cities in Taiwan (43). Patients with other forms of secondary hypertension, such as renovascular hypertension, Cushing’s syndrome, hyperthyroidism, and pheochromocytoma, were excluded from the study (44). All antihypertensive medications were discontinued for at least 21 days before conducting screening tests, with doxazosin or diltiazem administered as necessary to control markedly high blood pressure (45). PA diagnosis and subtype studies adhered to the standard protocols of TAIPAI and the aldosteronism consensus of Taiwan (46), including adrenal venous sampling (47) (see Supplemental Methods). Patients with uPA confirmed to have familial type I/GRA via long-range polymerase chain reaction were excluded from the analysis (48). Ethical approval (approval number 200611031R) was obtained from the institutional review board of the National Taiwan University Hospital. Written informed consent for clinical data collection and research use was obtained from all participants before enrollment in the study.

### Sample size calculation.

This case-control study was structured to maintain a type I error level of 0.05 and a type II error level of 0.05. The estimated number of patients with uPA was 56, and there were 22 control patients. We recruited double the minimum required number of participants to ensure robustness, resulting in a statistical power of 95% (Supplemental Methods).

### Parameter measurements.

We assessed the levels of total human FGF-23 using a kit from Cusabio Biotech, while the plasma levels of iFGF-23 and cFGF-23 were measured using kits from Immutopics.

The cFGF-23 assay detected both C-terminal fragment and full-length FGF-23, with FGF-23 measured in RU per milliliter. The coefficient of variation was below 10% for total FGF-23, 4.4% for iFGF-23, and 4.0% for cFGF-23. The lower limits of detection for total FGF-23, iFGF-23, and cFGF-23 were 6.2 pg/mL, 135 pg/mL, and 1.5 RU/mL, respectively.

We utilized a Human Plasma Klotho kit from Cusabio Biotech, with intra- and inter-assay imprecision values of less than 8.3% and 13.3%, respectively. The detection range of klotho was 0.156–10 ng/mL.

PAC was determined via radioimmunoassay using a commercial kit (Aldosterone Maia Kit, Adaltis Italia S.P.A.) (40), while PRA was measured by generating angiotensin I in vitro using a commercially available radioimmunoassay kit from DiaSorin (43). Additionally, 1,25(OH)_2_ vitamin D was measured using a DiaSorin radioimmunoassay assay kit, and total 25 OH vitamin D was measured using electrochemiluminescence (Elecsys, vitamin D total, Cobas, Roche Diagnostics). All assays were conducted according to the manufacturer’s protocol, with each measurement performed in duplicate.

### Outcome definitions.

We defined clinical outcomes according to the PASO consensus, requiring at least 2 follow-up visits. “Success” was determined by either complete clinical success or hypertension remission, indicating normalized blood pressure after adrenalectomy without the need for any antihypertensive medication (Supplemental Table 1) (49–51). The long-term composite outcomes of interest included all-cause mortality, stroke, cardiovascular events, or kidney events. Cardiovascular events encompassed nonfatal myocardial infarction, coronary artery bypass graft, nonfatal stroke, and positive findings in coronary angiography. New-onset kidney events were characterized by an eGFR of less than 60 mL/min/1.73 m^2^. To validate long-term outcome events, we cross-referenced TAIPAI records with the Taiwan National Health Insurance Research Database (52) (refer to Supplement Methods, *Outcomes of interest* section, for details).

### Cell culture study.

UMR-106 rat osteoblast-like osteosarcoma cells were provided by Sze-Kwan Lin from the Department of Dentistry at the National Taiwan University Hospital in Taipei, Taiwan. These cells were cultured in DMEM high-glucose medium supplemented with 10% fetal bovine serum and 1% penicillin-streptomycin at 37°C under a 5% CO_2_ atmosphere. To investigate the effects of aldosterone and a furin inhibitor on protein expression, the cells were seeded into 6-well plates at a density of 5 × 10^5^ cells per well. Subsequently, the cells were incubated with aldosterone at concentrations of 1, 10, and 100 nM, in the presence or absence of a 10 μM furin inhibitor (obtained from Cayman Chemical), for a duration of 12 hours. For Fam20C knockdown, shRNA was acquired from Applied Biological Materials. After 48 hours, the cells were transfected with 0.5 μg of scramble shRNA plasmid (piLenti-siRNA-GFP vector) or shFam20C plasmid using PolyJet transfection reagent from SignaGen Laboratories, following the manufacturer’s instructions.

### Western blot analysis.

Protein extraction from whole cell extracts was performed using RIPA buffer (composed of 50 mM Tris base pH 8, 150 mM NaCl, 1% NP-40, and 0.10% SDS) supplemented with a protease inhibitor (obtained from Roche Diagnostics). Following extraction, the cell lysates were centrifuged at 16,000*g* for 15 minutes at 4°C and then transferred to PVDF membranes, as described previously (53). For Western blot analysis, polyclonal anti-furin antibody (sc-20801, obtained from Santa Cruz Biotechnology); mouse monoclonal antibodies targeting iFGF-23 and cFGF-23 (MAB26291, acquired from R&D Systems, Bio-Techne), with the distinction between the two made based on their molecular sizes on a gel; rabbit polyclonal FAM20C antibody (25395-1-AP, sourced from Proteintech); and rabbit polyclonal anti-GAPDH antibody (MAB374, obtained from MilliporeSigma) were utilized. To validate the detection capabilities of the MAB26291 antibody, we conducted additional experiments using recombinant cFGF-23 (Supplemental Figure 7).

### Aldosterone-infused mice and PTH inhibitor (cinacalcet) administration.

Male C57BL/6 mice, aged 8 to 10 weeks, were obtained from the National Laboratory Animal Center in Taipei, Taiwan. Three groups were established, each consisting of 4 mice: (a) nephrectomy (Nx)-vehicle (control) group, undergoing Nx surgery followed by oral gavage treatment with normal saline; (b) aldosterone infusion-vehicle group, receiving aldosterone infusion followed by oral gavage treatment with normal saline; and (c) aldosterone infusion-PTH inhibitor (cinacalcet) group, undergoing aldosterone infusion followed by oral gavage treatment with cinacalcet (15 mg/kg body weight daily) (Supplemental Methods).

### Aldosterone’s influence on FGF-23 transition via the Fam20C pathway.

For in vitro kinase assay, cell lysates were extracted from UMR-106 cells, with or without prior aldosterone treatment (15). Kinase assays were conducted using 10 μg of cell lysates and 10 μL of Phos-ag agarose resin (Wako Fujifilm). The mixtures were incubated at 4°C for 1 hour in 500 μL binding buffer following the manufacturer’s instructions. Phos-agarose immunoprecipitation was performed, and phosphoproteins were separated through 15% SDS-PAGE and detected via Western blot analysis utilizing an anti–FGF-23 antibody (54).

### Molecular docking of FAM20C with aldosterone.

Molecular docking was carried out to elucidate the interactions between FAM20C and aldosterone using the SwissDock server and EADock. The structure of FAM20C was adapted from the 3-dimensional structure reported previously (PDB: 5YH3) (22). The binding modes were generated either locally or in the vicinity of all target cavities, and their energies were estimated simultaneously on a grid using Chemistry at HARvard Molecular Mechanics (55). The binding modes with the most favorable energies were evaluated using fast analytical continuum treatments of solvation and clustered.

### Statistics.

Statistical analyses were performed using SPSS software version 20 (IBM), R software version 3.2.2 (Free Software Foundation, Inc.), and MedCalc Statistical Software version 15.11.3 (MedCalc Software bvba; https://www.medcalc.org; 2015). Continuous data were presented as mean ± SD and compared using the 2-tailed unpaired *t* test. Categorical data were expressed as number (percentage) and compared using the χ^2^ or Fisher’s exact test. Continuous variables in the baseline characteristics were expressed as mean ± SD. For Tables 1 and 2, the differences among groups were compared using the Mann-Whitney *U* test for continuous variables; for categorical variables, the χ^2^ test was used. Normal distribution was achieved by transforming skewed variables such as iFGF-23, cFGF-23, PAC, and ARR. For Figure 1, repeated measures 1-way ANOVA, followed by post hoc Bonferroni’s correction, was employed for multiple comparisons.

Receiver operating characteristic curves were generated, and areas under the curves were calculated to assess the performance of candidate biomarkers. Factors including plasma cFGF-23 and some interactions, such as aldosterone profiles, were included in a selected variable list to predict hypertension remission. The significance levels for entry and for stay were set at 0.15. The final logistic regression model or Cox proportional model was identified manually by dropping covariates with *P* > 0.05 until all regression coefficients were significantly different from 0. The variables included in the final model were age, sex, body mass index, potassium level, aldosterone, renin, hypertension duration, plasma cFGF-23 level, and systolic and diastolic blood pressure.

To identify the effects of cFGF-23 in individual patients, a GAM with spline incorporating participant-specific (longitudinal) random effects was plotted with adjustments for other clinical parameters to predict clinical remission (56, 57). Nonlinear effects of continuous covariates were explored using multiple GAMs to determine appropriate cutoff points for concretizing cFGF-23. The optimal cutoff value was defined as the point where the log odds equaled 0 (58).

All *P* values are 2 sided and considered statistically significant when less than 0.05.

### Study approval.

This research adhered to the guidelines stipulated in the Human Tissue Act and received approval from our Institutional Ethical Committee (National Taiwan University Hospital, Taipei, Taiwan; approval number: 200611031R), with written informed consent obtained from all participants. Animal procedures were conducted in compliance with an approved protocol from the National Taiwan University Institutional Animal Care and Use Committee (protocol number: 20210381).

### Data availability.

Values for all data points found in graphs are in the Supporting Data Values file.

## Author contributions

VCW and KYP participated in designing research studies with statistical analysis and wrote the manuscript. KYP, TIC, CYS, HWL, CKC, YHL, and JSC participated in conducting experiments and acquiring data. VCW, JSC, and CKC participated in analyzing data and providing reagents.

## Supplementary Material

Supplemental data

Unedited blot and gel images

Supporting data values

## Figures and Tables

**Table 1:**
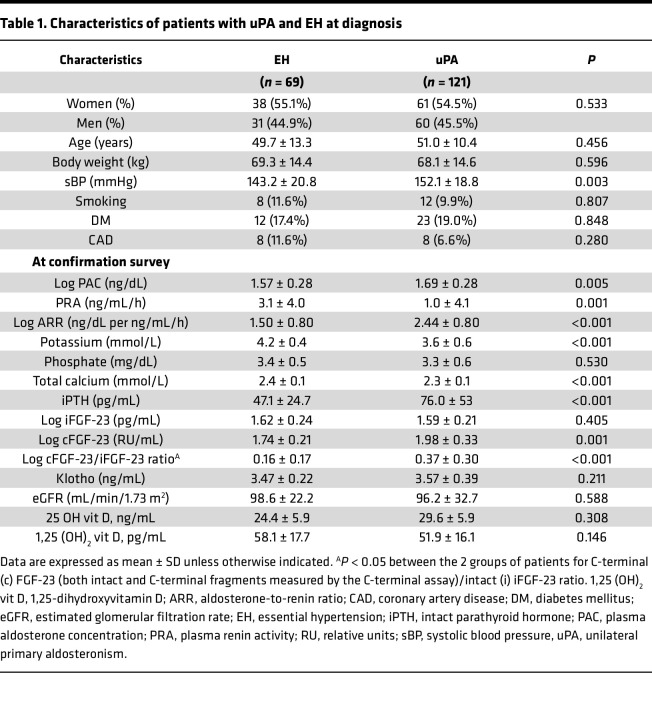
Characteristics of patients with uPA and EH at diagnosis

**Table 2:**
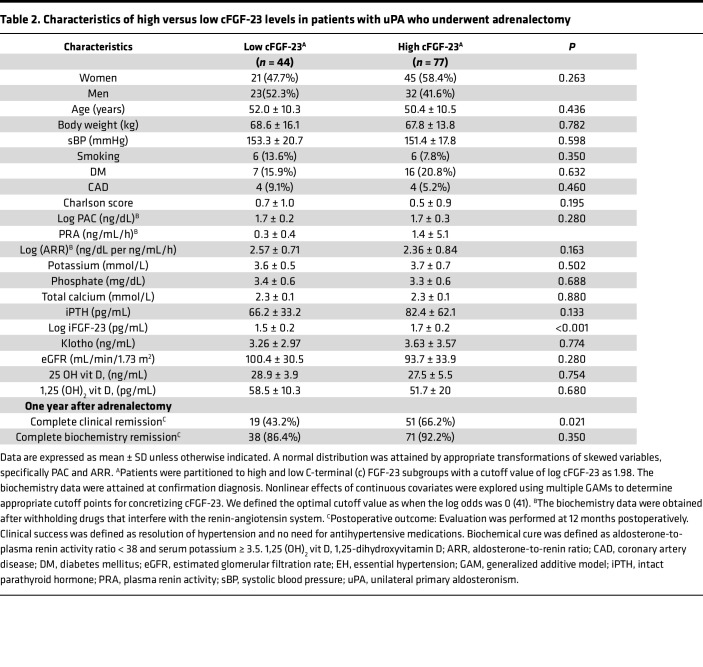
Characteristics of high versus low cFGF-23 levels in patients with uPA who underwent adrenalectomy

## Version 1 02/24/2025

Electronic publication
