# Supplementary material for: C-terminal FGF-23 production coupling with aldosterone via FAM20C and predicting cardiovascular events in primary aldosteronism
Source: JCI Insight. 2025 Feb 24;10(4):e166461. doi: 10.1172/jci.insight.166461 (PMC11949054; doi:10.1172/jci.insight.166461)
Supplement: Supplemental data [file jciinsight-10-166461-s065.pdf]

Supplementary data

Standard TAIPAI protocol and Aldosteronism Consensus in Taiwan

Patients were enrolled from the following hospitals:

This study included two medical centers (National Taiwan University Hospital (NTUH), Taipei, Taiwan; Taipei University Hospital, Taipei, Taiwan) and five regional hospitals (Cardinal Tien Hospital, New Taipei City, Taiwan; Taipei Tzu Chi Hospital, New Taipei City, Taiwan; Yun- Lin Branch of NTUH, Douliou City, Taiwan; Hsin-Chu Branch of NTUH, Hsin-Chu City, Taiwan; Zhongxing Branch of Taipei City Hospital, Taipei, Taiwan)(1).

Material and methods

Ethical approval (approval number 200611031R) was obtained from the institutional review board of the National Taiwan University Hospital. Written informed consent for clinical data collection and research use was obtained from all participants before enrollment in the study.

Statistics

E-values

We computed E-values using the methodology proposed by Vander Weele and Ding.[(2)] Specifically, the E-values quantify what the risk ratio would need to be for unmeasured confounders to explain away the observed associations of cFGF-23 level to all-cause mortality and cardiovascular events in the present study.

Sample size determination

Because this study is a pilot study, the sample size calculated based on data from the previously reported study from pediatric patients with secondary hypertension.(3) We assumed the mean value ± standard deviation of plasma FGF-23 according to hypertensive patients and normotensive controls.

This was case-control study designed to have a type I error level of 0.05 and type II error level of 0.05 to detect a mean difference of 7.27 and standard deviation of patients (N=98) is 12.16, and normotensive control (n=37) is 5.22. The minimum required number of hypertensive patients is 56, while controls is 22 and the power is 95%. (MedCalc v19.1 (MedCalc Software bvba, Ostend, Belgium).

		Type I Error - Alpha			
		0.20	0.10	0.05	0.01
Type II Error - Beta	0.20	20 + 8	27 + 11	35 + 14	52 + 20
	0.10	28 + 11	37 + 14	46 + 18	65 + 25
	0.05	37 + 14	47 + 18	**56 + 22**	77 + 30
	0.01	55 + 21	67 + 26	78 + 30	103 + 39

*Left number of study group+ right number of control group.

Adrenalectomy

Adrenalectomy was performed via lateral transperitoneal laparoscopic approach by experienced surgeons. Adrenal tumors removed via the surgery were fresh-frozen and stored at –80°C until further examination.

Our standard protocol to identify primary aldosteronism (PA) and functional lateralization:

The diagnosis of primary aldosteronism was established in hypertensive patients on the basis of the following criteria(1-4) (Fig S1):

<u>Confirmation</u>

Fulfillment of the following three conditions confirmed a diagnosis of PA:
(1) autonomous excess aldosterone production evidenced with an aldosterone-renin ratio (ARR) > 35; (2) a TAIPAI score larger than 60%; (3) post-saline loading PAC > 16 ng/dL, or PAC/PRA > 35 (ng/dL)/(ng/mL/h) shown in a post-captopril/losartan test. (Abbreviations: PAC, plasma aldosterone concentration; PRA, plasma renin activity) (1).

The probability of PA (TAIPAI score) was equal to:
= 1 + $e^{-\beta}$; where β = (PAC [ng/dl] × [0.063]) + PRA [ng/ml/h] × [−0.205]) + ([ARR × 0.001] BMI [kg/m^2] × [0.067]) + (Male × [−0.738] + SK [mmol/l] × [−1.512]) + (eGFR [ml/min/1.73 m^2] × [0.017]) + ([propensity score] × [−0.539] + [1.851])

<u>Unilateral PA</u>

Unilateral PA (aldosterone producing adenoma) was identified on the basis on the following four criteria(1): (1) Confirmed PA; (2) an adrenal adenoma or hyperplasia evidenced with a CT or MRI scan [6]; (3) lateralization of aldosterone secretion with adrenal vein sampling (AVS) on the imagine finding side;

Aldosterone producing adenoma/ nodules (APA/APN) is further confirmed after adrenalectomy:
(4) pathologically proven a CYP11B2 adenoma or (multiple) aldosterone-producing nodule / micronodule at immunohistochemistry according to the HISTALDO consensus(5-6) after adrenalectomy, and subsequent emergence of biochemical correction.

Selectivity and lateralization indices of AVS without stimulation tests
The selectivity index (SI) is defined as the ratio of the sampled cortisol concentration of each adrenal vein to that of the peripheral vein. The lateralization index (LI) is defined as the ratio of the aldosterone/cortisol concentration on the dominant side to that on the contralateral side. Successful AVS is defined as an SI value ≥2.0 bilaterally. After confirming successful bilateral AVS, lateralization of the PA was determined by an LI value ≥2.0.

Total FGF-23 ELISA kits (Cusabio Biotech, Wuhan, China)
coefficient of variation was < 10%
Precision
Intra-Assay: CV< 8 %
Inter-Assay: CV<10 %
Range: 3.12pg/ml-200pg/ml
Sensitivity: 0.78ng/ml

iFGF-23 ELISA kits (Immutopics; San Clemente, CA, USA)
coefficient of variation was < 4.4%
Precision
Intra-Assay: CV< 4.1%
Inter-Assay: CV<9.1 %
Range: 22-2253 pg /ml
Sensitivity: 1.5pg/ml

cFGF-23 ELISA kits (Immutopics; San Clemente, CA, USA)
coefficient of variation was < 4.0%
Precision
Intra-Assay: CV< 2.4 %
Inter-Assay: CV<4.7 %
Range: 20-1430 RU /ml

Sensitivity: 1.5 RU /ml

Plasma Klotho kit (Cusabio Biotech, Wuhan)
coefficient of variation was < 4.0%
Precision
Intra-Assay: CV< 8 %
Inter-Assay: CV<10 %
Range: 0.156-10ng/ml
Sensitivity: 0.039ng/ml

Sample Preparation

- Plasma was stored for a short periodat 2 - 8 °C or aliquoted at -20 °C (≤1 month) or -80 °C (≤ 3 months). Repeated freeze-thawcycles was avoided. Prior to assay, the frozen samples were slowly thawed and centrifuged to remove precipitates.
- We diluted the sample with appropriate folds of Sample Dilution Buffer into one Standard tube to make the diluted target protein concentration fall in the optimal detection range of the kit.

Biochemistry Measurements

We collected baseline patient data, including age, sex, weight, height, blood pressure, past medical history, and biochemistry profile. Weight and height were used to calculate body mass index (BMI). Estimated glomerular filtration rate (eGFR) was calculated using the Modification of Diet in Renal Disease equation.

All anti-hypertensive medications were discontinued for at least 21 days before performing these tests, although diltiazem and/or doxazosin were used to manage high blood pressure when needed.

Plasma aldosterone concentration and plasma renin activity were measured using radioimmunoassay commercial kits manufactured by Cisbio Bioassays, Codolet, France, and Beckman Coulter, Prague, Czech Republic, respectively.

Measurement of furin-like enzyme activity

In this study, cell lysates were prepared following the established protocol by (7). The

procedure began with the thawing of cell lysates on ice, which were subsequently subjected to a 2-fold dilution using 5× lysis/reaction buffer. Next, 20 µl of the prepared lysates were dispensed into black opaque 96-well plates (Greiner Bio-One, Austria), with each well containing 70 µl of MilliQ water (Millipore, MA, USA). All experimental samples were assayed in triplicate. The plates were then subjected to a 15-minute incubation at 37 °C within a Victor™ X3 plate reader (Perkin Elmer, MA, USA). Following this incubation, 10 µl of 1 mM furin fluorogenic substrate, pre-warmed for 30 minutes at 37 °C and protected from light, were added. Fluorescence intensity was immediately measured, utilizing excitation at 355 nm and emission at 460 nm, each reading performed over 1.0 seconds. Subsequent measurements were taken typically at 3-minute intervals, spanning up to 4 hours. Black FluoroNunc 96-well plates with MaxiSorp surface (Nunc, Denmark) were utilized for overnight coating with 50 µl/well of affinity-purified goat polyclonal anti-human furin antibody (R&D Systems, MA, USA) at a concentration of 10 µg/ml in 50 mM Na2CO3, pH 9.6, at room temperature, while being shielded from light. Finally, 20 µl of 1 mM pre-warmed furin fluorogenic substrate were introduced into each well. This assay procedure was conducted in triplicate for all samples.

Cell Culture and Aldosterone Treatment of IDG-SW3 Cells

The murine osteocyte cell line, IDG-SW3, was procured from Kerafast (Boston, MA, USA) and cultivated following the manufacturer's instructions. Briefly, the cells were cultured in MEMα medium (Thermo Fisher Scientific, Waltham, MA, USA) supplemented with 10% FBS, 1% penicillin/streptomycin, and 50 U/mL IFN-γ. Culturing was conducted on type-I collagen-coated dishes at 33 °C. Osteogenic differentiation was induced by seeding cells on type I collagen-coated plates and culturing them in a medium containing 50 µg/ml ascorbic acid and 4 mM β-glycerophosphate, while excluding IFN-γ. After 34 days of differentiation, the osteocytes were treated with aldosterone at concentrations of 1, 10, and 100 nM for a duration of 24 hours.

Aldosterone-Infused Mice and PTH Inhibitor (Cinacalcet) Administration

Male C57BL/6 mice, aged 8 to 10 weeks, were procured from the National Laboratory Animal Center (Taipei, Taiwan). Uninephrectomy (Nx) was performed under anesthesia using a cocktail of 40 mg/kg Zoletil and 12 mg/kg xylazine via intraperitoneal injection on day 0. Simultaneously, the mice were randomized for subcutaneous implantation of an osmotic mini-pump (model 2004, Alzet). The mini-pumps contained either aldosterone (500 µg/kg/day, dissolved in 5% EtOH) or vehicle (5% EtOH) and were administered for a period of 28 days. Three distinct

groups, each comprising 3 mice, were established: (1) Nx-vehicle (control) group, involving Nx surgery followed by oral gavage treatment with normal saline; (2) Aldosterone infusion-vehicle group, in which aldosterone infusion was followed by oral gavage treatment with normal saline; (3) Aldosterone infusion-PTH inhibitor (cinacalcet) group, where aldosterone infusion was followed by oral gavage treatment with cinacalcet (15 mg/kg body weight daily).

Measurement of Plasma Biomarkers
Plasma levels of intact and C-terminal forms of FGF-23 were determined utilizing the Mouse/Rat FGF-23 (Intact) and (C-Term) ELISA kit (Quidel, San Diego, CA, USA). Intact parathyroid hormone (iPTH) levels were measured through radioimmunoassay (RIA) on the PerkinElmer automatic gamma counter 1470 wizard series. Plasma ionized calcium (iCa) levels were determined using the Cobas B 221 blood gas and electrolyte analyzer (Roche Diagnostics Ltd., Taiwan).

Recombinant cFGF-23 and MAB26291 Detection

To validate the detection capabilities of the MAB26291 antibody, we conducted additional experiments using recombinant cFGF-23. Specifically, we utilized a synthetic rat C-terminal FGF23 peptide (residues Ser180-Val251), which was obtained from Motif Biotechnology (Suzhou, China). The results confirmed that the MAB26291 antibody was able to detect the precise location of the C-terminal FGF23 fragments. This finding is consistent with the detection capability of the Immutopics ELISA antibody(8), further ensuring the accuracy and reliability of our assay.

<u>Outcome evaluation</u>

Supplementary Table 1. Assessment of clinical and biochemical outcomes after unilateral adrenalectomy according to the PASO criteria. At 6 to 12 months postoperatively, complete clinical success was defined as normalized blood pressure without the use of antihypertensives. Complete biochemical success was defined as normalized serum potassium levels (≥3.5 mmol/L) and aldosterone-to-renin ratio.

Supplementary table 1

**OUTCOME**	**COMPLETE (Remission)**	**PARTIAL (Improvement)**	**ABSENT (Persistence)**

CLINICAL SUCCESS	Normal BP without antihypertensive medication	Same BP as before surgery with less antihypertensive medication or decreased BP with the same or less antihypertensive medication	Unchanged or increased BP with the same or increased antihypertensive medication
BIOCHEMICAL SUCCESS	Correction of hypokalaemia and normalization of ARR or suppression of aldosterone secretion in post-surgical confirmatory test (if ARR elevated)	Correction of hypokalaemia and elevated ARR with ≥ 50% reduction baseline PAC and/or elevated but improved post-surgical CCT	Persistent hypokalaemia and/or persistent elevation of ARR with failure to suppress aldosterone secretion in post-surgical CCT
INITIAL ASSESSMENT	Initial outcome assessment within 3 months post-surgery for adjustment of anti-HT medication and correction of hypokalaemia or hyperkalaemia if necessary		
FINAL ASSESSMENT	Final outcome assessment at 6–12 months after adrenalectomy		
ASSESSMENT INTERVAL	Reassessment of outcomes at yearly intervals for an indefinite period to exclude persistence or reoccurrence of disease		

ARR= aldosterone-to-renin ratio; BP= blood pressure; CCT=captopril challenging test; PAC= plasma aldosterone concentrations

Results

Supplementary table 2

Factor associated with cardiovascular composite outcomes* by Cox proportional Hazard model.

**Parameter**	**p**	**Hazard ratio**	**95% CI**	
Age (years)	0.746	0.994	0.957	1.032
Gender (Male)	0.051	2.426	0.996	5.913
BMI (Kg/M2)	0.651	1.023	0.926	1.131
Potassium (mmol/L)	0.232	0.672	0.350	1.290
sBP (mmHg)	<.0001	1.061	1.032	1.091
dBP (mmHg)	0.043	0.959	0.922	0.999
PAC (ng/dL)	0.725	0.998	0.985	1.010
PRA (ng/ml/hr)	0.580	0.796	0.355	1.784
Hypertension duration (yrs)	0.036	1.095	1.057	1.132
Log [plasma cFGF-23] > 1.98	0.002	4.176	1.714	10.176

* The long- term cardiovascular composite outcome were the occurrence of either all-cause mortality, stroke, cardiovascular events or new-onset chronic kidney disease.
Abbreviations: BMI, body mass index, PAC, plasma aldosterone concentration; PRA, plasma renin activity; sBP, systolic blood pressure.

Outcomes of interest

All-cause mortality was the primary outcome. Our secondary outcomes included de-*novo* (incident) MACE, new onset of kidney disease after the index date of PA confirmatory diagnosis. MACE was the incidence of new-onset coronary events including non-fatal myocardial infarction (MI), coronary artery bypass graft (CABG), stroke and coronary angiography.
For corroborating long-term events, we further validated TAIPAI records with Taiwan National Health Insurance Research Database (NHIRD). The Taiwan NHI is a nationwide insurance program that covers ambulatory visits, hospital admissions, prescriptions, interventional procedures and disease profiles for over 99% of the population in Taiwan (23.12 million in 2009). (9-12) The ICD-9 code of MI at hospitalization has high accuracy, as validated by previous research. (10-13) The

records of CABG and angiography are also very reliable because they were constructed on the basis of NHI procedure codes that were tied to the NHI reimbursement system with regular auditing. (10) The diagnosis of stroke was an outcome of interest with validation from both radiographic image reports and ICD-9 diagnosis codes in this study and has been well attested. (14-15) New-onset kidney disease was defined as estimated glomerular filtration rate < 60 ml/min/1.73m^2.

Supplementary Figure

Supplementary **Figure 1.**The correlation of *various* parameters level. The distribution of each variable is shown on the diagonal. On the bottom of the diagonal are the bivariate scatter plots. On the top of the diagonal are the value of the correlation. Blue color depicts positive correlation while red color depicts negative correlation. All the circle symbols of p<0.05.

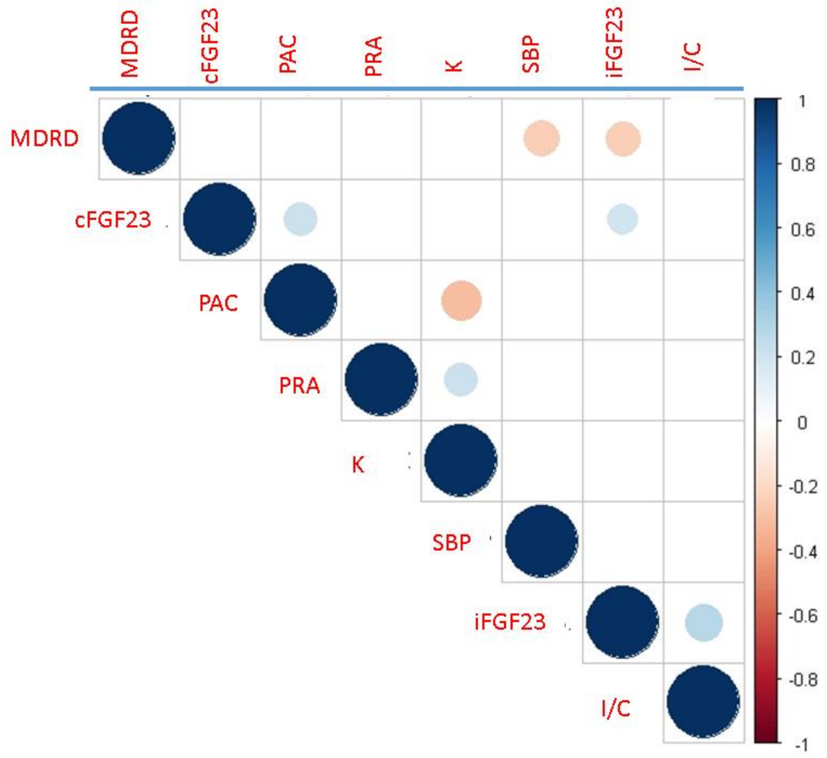

Supplementary **Figure 2.** Adjusted spline of the (A) cFGF-23 (B) iFGF-23 against log plasma aldosterone level of all uPA patients. The probability of outcome event was constructed with plasma cFGF-23 level that have an average of zero over the range of

the data, i.e., Log PAC = 1.98. The dashed lines indicate approximated point-wise 95% CI.

A

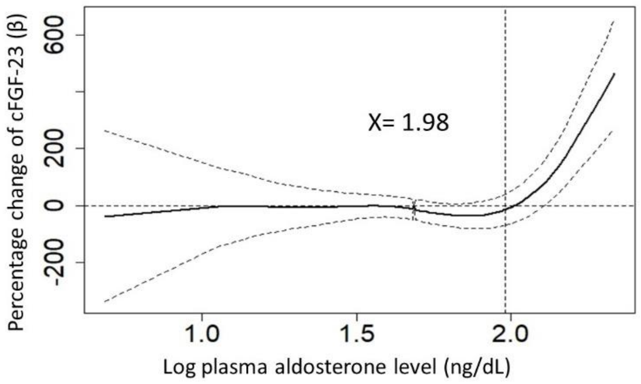

B

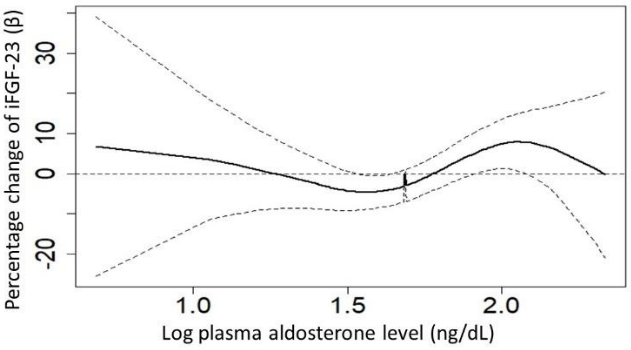

Supplementary Figure 3.

Aldosterone treatment increase C-terminal form of FGF-23 level in differentiated IDG-SW3 cells

To validate the effect of aldosterone on FGF-23 in another osteocyte cell line, IDG-SW3 cells. Aldosterone treated differentiated IDG-SW3 cells were analyzed for FGF-23 protein level by Western blot analysis. Results showed that aldosterone treatment increased level of cFGF-23 in IDG-SW3 osteocytes but not Furin, Galnt3 or Fam20C.

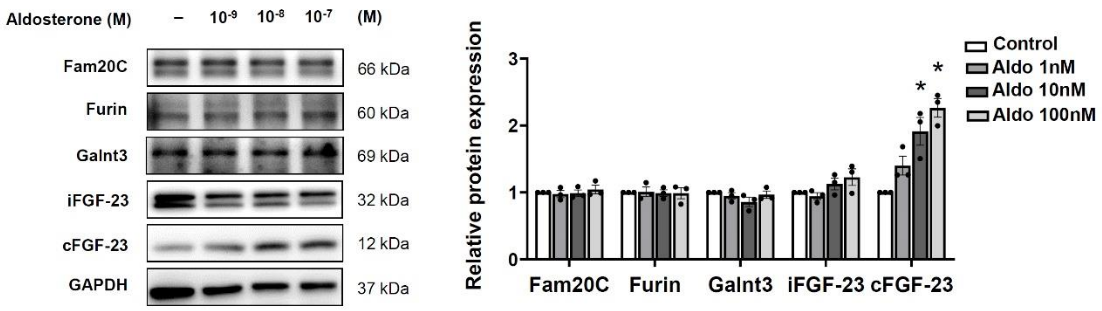

Effect of aldosterone on level of intact and c-terminal form of FGF-23 in differentiated IDG-SW3 cells. Western blot analysis showed the level of intact and c-terminal form of FGF-23 of 34 days differentiated IDG-SW3 cells after treatment with aldosterone for 24 hours. Quantification of the bands on Western blot were performed. The results were based on three independent experiments. Data were compared by one-way ANOVA with Bonferroni post-hoc correction for multiple comparisons.

*p<0.05, **p<0.01,. Graphical data are shown as mean +/- S.E.M.

Supplementary **Figure 4.**

Cell lysates of UMR-106 were generated following aldosterone infusions at varying concentrations (ranging from 10^{-6} to 10^{-8}M). Notably, the aldosterone failed to induce an increase in furin-like enzyme activity across different doses of aldosterone infusion. Subsequently, a furin inhibitor was introduced as a negative control, and intriguingly, it was observed that the furin inhibitor successfully restored furin activity when co-cultured with aldosterone.

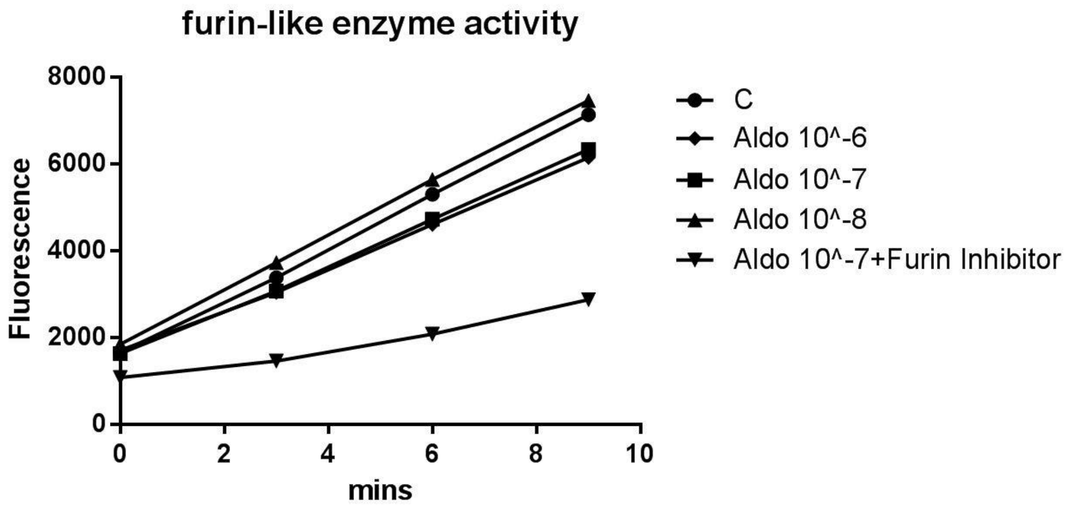

Supplementary Figure 5

Aldosterone-infused mice exhibit higher circulating cFGF-23 levels, which cannot be reversed by PTH inhibitor (cinacalcet) treatment.

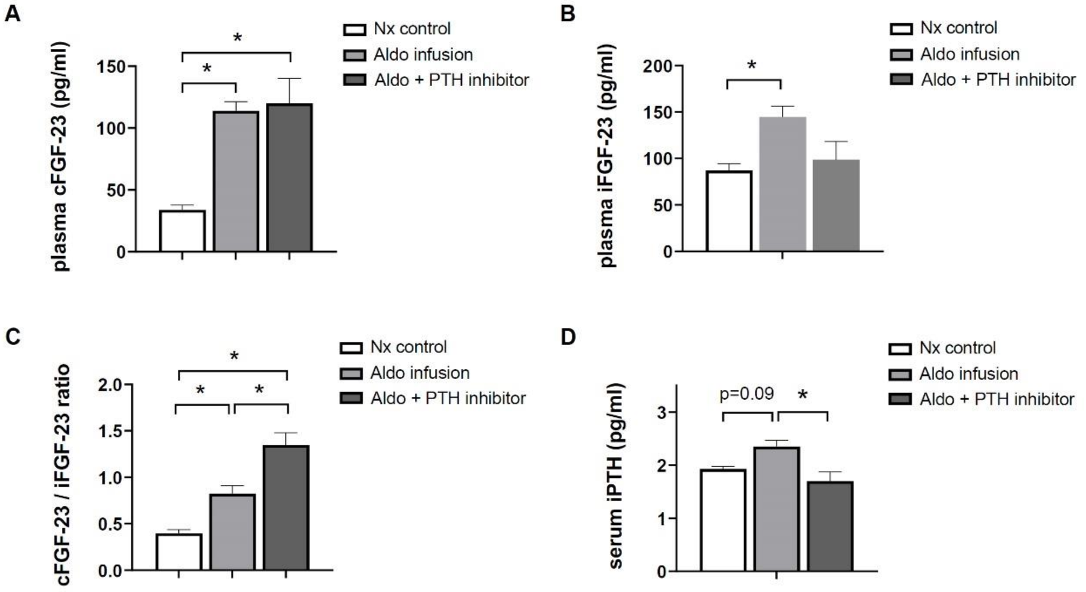

Figure Legend:

Effect of PTH inhibitor (cinacalcet) on circulating levels of cFGF-23 and iPTH in 28-day aldosterone-infused mice.

(A) Plasma cFGF-23 levels increased after aldosterone infusion and were not restored by the PTH inhibitor ($p<0.05$).

(B) Plasma iFGF-23 levels increased after aldosterone infusion and were restored by treatment with the PTH inhibitor ($p<0.05$).

(C) The c/i FGF23 ratio approximated 1 after aldosterone infusion and increased following PTH inhibitor treatment ($p<0.05$).

(D) After aldosterone infusion, the iPTH level was restored following treatment with the PTH inhibitor ($p<0.01$).

Experiments were conducted in duplicates with four mice for each group (treated or control). Data were compared by one-way ANOVA with Bonferroni post-hoc correction for multiple comparisons. Data are represented as mean ± SEM.

Supplementary Figure 6

An illustrative diagram depicting preoperative cFGF-23 could predict hypertension remission preoperative plasma cFGF-23 level could predict hypertension remission and a greater risk of long-term mortality and incident cardiovascular events in patients with unilateral primary aldosteronism. The over-production of cFGF-23 is due to an increase in iFGF-23 through the augmentation of aldosterone-induced FAM20C activity and altered the binding ability.

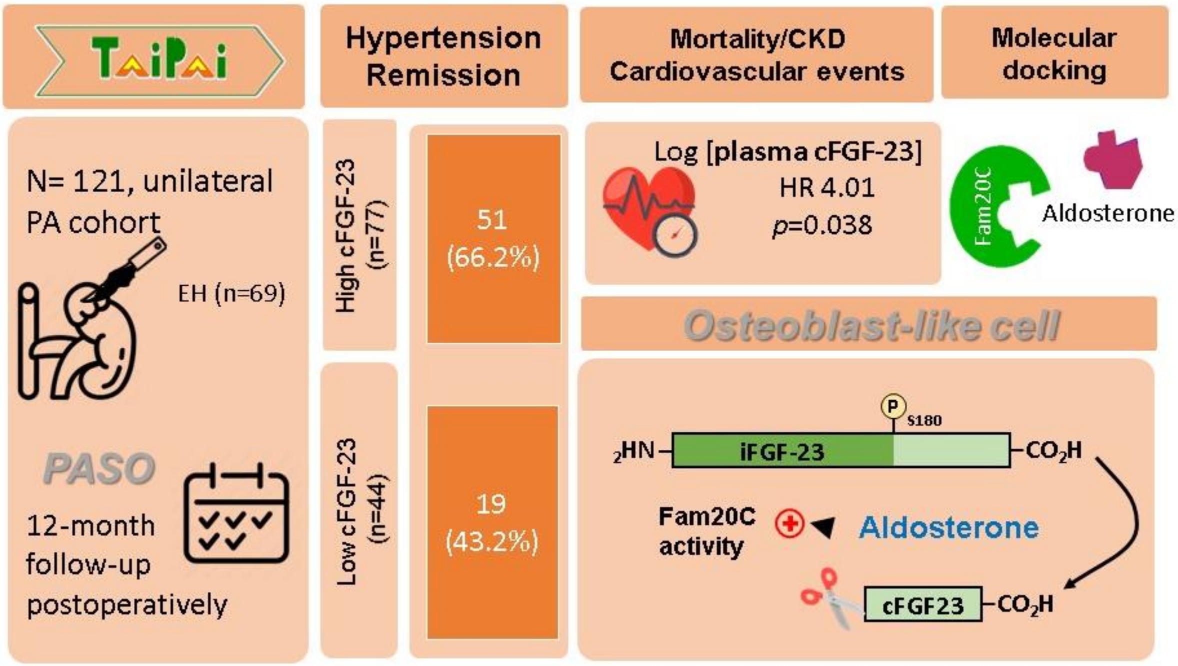

Abbreviations: cFGF-23, carboxyl terminal FGF-23; CKD, chronic kidney disease; Fam20C, family with sequence similarity 20, member C; EH, essential hypertension; iFGF-23, intact FGF-23; uPA, unilateral primary aldosteronism

Supplementary Figure 7

Detection of recombinant cFGF-23 using two different antibodies. The synthetic rat C-terminal FGF23 peptide (residues Ser180-Val251), which was obtained from Motif Biotechnology (Suzhou, China). Western blot analysis of 30 ng of recombinant cFGF-23 was performed using (left) the mouse FGF-23 antibody (MAB26291) from R&D Systems and (right) the mouse FGF-23 antibody (C-terminal specific, catalog number 40-6320) from Immutopics. Both antibodies detect the C-terminal region of FGF-23, with bands appearing near the expected molecular weight for cFGF-23. These results confirm the ability of MAB26291 to recognize the C-terminal region, consistent with

detection by the Immutopics antibody. Molecular weight markers are indicated in kilodaltons (kDa) on the left of each blot.

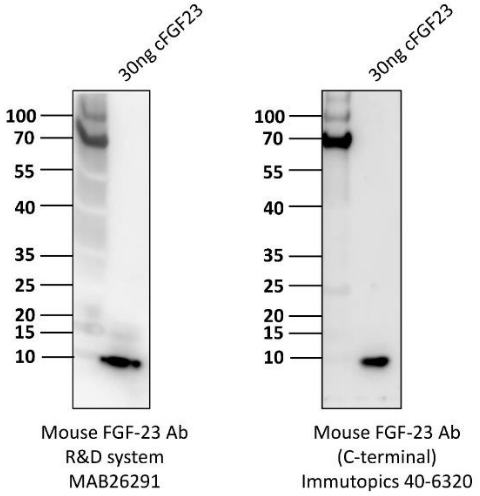

Supplemental Acknowledgments

The PAC and TAIPAI included two medical centers (National Taiwan University Hospital (NTUH), Taipei, Taiwan; Taipei University Hospital, Taipei, Taiwan) and five regional hospitals (Cardinal Tien Hospital, New Taipei City, Taiwan; Taipei Tzu Chi Hospital, New Taipei City, Taiwan; Yun- Lin Branch of NTUH, Douliou City, Taiwan; Hsin-Chu Branch of NTUH, Hsin-Chu City, Taiwan; Zhongxing Branch of Taipei City Hospital, Taipei, Taiwan). The coprincipal investigators in each sites are (Http://doi.org/10.6084/m9.figshare.21669929)

Vin-Cent Wu[1], Tai-Shuan Lai[1], Shih-Chieh Jeff Chueh[1], Shao-Yu Yang[1], Kao-Lang Liu[1], Chin-Chen Chang[1], Bo-Chiag Lee[1], Shuo-Meng Wang[1], Kuo-How Huang[1], Po-Chih Lin[1], Yen-Hung Lin[1], Chi-Sheng Hung[1], Lian-Yu Lin[1], Shih-Cheng Liao[1], Ching-Chu Lu[1], Chieh-Kai Chan[1], Leay-Kiaw Er[2], Ya-Hui Hu[2], Che-Hsiung Wu[2], Yao-Chou Tsai[2], Zheng-Wei Chen[2], Chien-Ting Pan[3], Che-Wei Liao[4], Cheng-Hsuan Tsai[5], Yi-Yao Chang[6], Chen-Hsun Ho[7], Wei-Chieh Huang[8], Ying-Ying Chen[9]

1. National Taiwan University Hospital, Taipei, Taiwan;
2. Taipei Tzu-Chi Hospital, Buddhist Tzu Chi Medical Foundation, Taipei, Taiwan;

3. Department of Internal Medicine, National Taiwan University Hospital Yun-Lin Branch, Yun-Lin, Taiwan;
4. Department of Medicine, National Taiwan University Cancer Center, Taipei, Taiwan;
5. Department of Internal Medicine, National Taiwan University Hospital Jin-Shan Branch, New Taipei City, Taiwan;
6. Department of Cardiovascular Medicine, Far Eastern Memorial Hospital, New Taipei City, Taiwan
7. Shin Kong Wu Ho-Su Memorial Hospital, Taipei, Taiwan;
8. Taipei Veterans General Hospital, Taipei, Taiwan;
9. MacKay Memorial Hospital, Taipei, Taiwan.

References

1. Wu VC, et al. Case detection and diagnosis of primary aldosteronism - The consensus of Taiwan Society of Aldosteronism. *J Formos Med Assoc.* 2017;116(12):993-1005.
2. VanderWeele TJ, and Ding P. Sensitivity analysis in observational research: introducing the E-value. *Ann Intern Med.* 2017;167(4):268-274.
3. Lin Y, et al. Plasma Fibroblast Growth Factor 23 Is Elevated in Pediatric Primary Hypertension. *Front Pediatr.* 2019;7:135.
4. Wu VC, et al. Prevalence and clinical correlates of somatic mutation in aldosterone producing adenoma-Taiwanese population. *Sci Rep.* 2015;5:11396.
5. Williams TA, et al. International Histopathology Consensus for Unilateral Primary Aldosteronism. *The Journal of clinical endocrinology and metabolism.* 2021;106(1):42-54.
6. Wu VC, et al. Subtypes of Histopathologically Classical Aldosterone-Producing Adenomas Yield Various Transcriptomic Signaling and Outcomes. *Hypertension.* 2021:HYPERTENSIONAHA12118006.
7. Bourne GL, and Grainger DJ. Development and characterisation of an assay for furin activity. *J Immunol Methods.* 2011;364(1-2):101-108.
8. Braithwaite V, et al. Intact fibroblast growth factor 23 and fragments in plasma from Gambian children. *Osteoporos Int.* 2013;24(3):1121-1124.
9. Wu PC, et al. Long-term risk of upper gastrointestinal hemorrhage after advanced AKI. *Clin J Am Soc Nephrol.* 2015;10(3):353-362.
10. Wu VC, et al. Long-term risk of coronary events after AKI. *J Am Soc Nephrol.* 2014;25(3):595-605.
11. Wang WJ, et al. The impact of acute kidney injury with temporary dialysis on the risk of fracture. *Journal of bone and mineral research : the official journal of the American Society for Bone and Mineral Research.* 2013;29(3):676-684.
12. Wu PC, et al. Pentoxifylline Decreases Dialysis Risk in Patients With Advanced Chronic Kidney Disease. *Clinical pharmacology and therapeutics.* 2015;98(4):442-449.
13. Lu CC, et al. Prognostic value of semiquantification NP-59 SPECT/CT in primary aldosteronism patients after adrenalectomy. *Eur J Nucl Med Mol Imaging.* 2014;41(7):1375-1384.
14. Chen HJ, et al. Influence of metabolic syndrome and general obesity on the

risk of ischemic stroke. *Stroke.* 2006;37(4):1060-1064.
15. Wu VC, et al. The impact of acute kidney injury on the long-term risk of stroke. *Journal of the American Heart Association.* 2014;3(4):e000933.
