# Supplementary material for: C-terminal FGF-23 production coupling with aldosterone via FAM20C and predicting cardiovascular events in primary aldosteronism
Source: JCI Insight. 2025 Feb 24;10(4):e166461. doi: 10.1172/jci.insight.166461 (PMC11949054; doi:10.1172/jci.insight.166461)

MS TITLE: Carboxyl-terminal FGF-23 production coupling with aldosterone via FAM20C and predicting cardiovascular events in primary aldosteronism

Full unedited gel for each Figure

Figure 3A

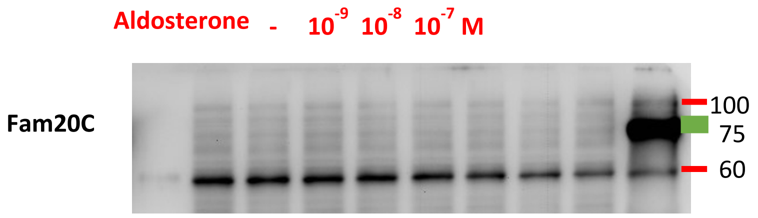

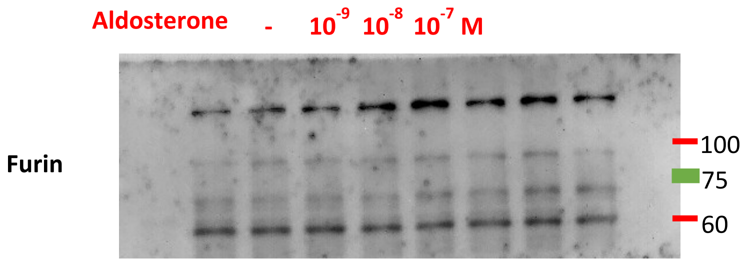

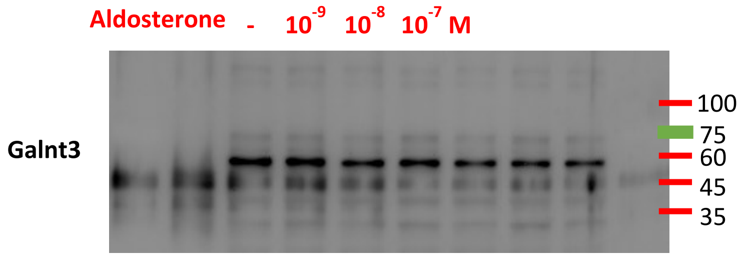

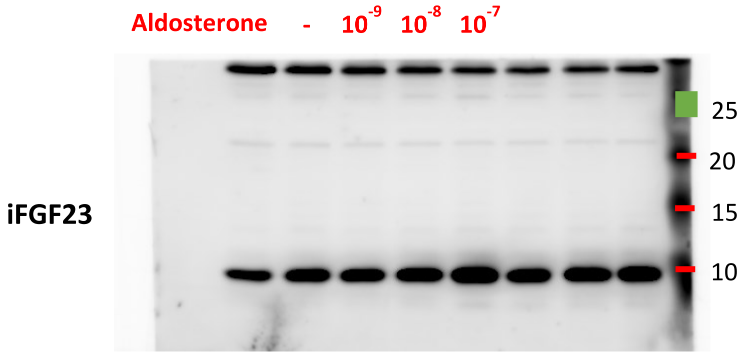

Figure 3A

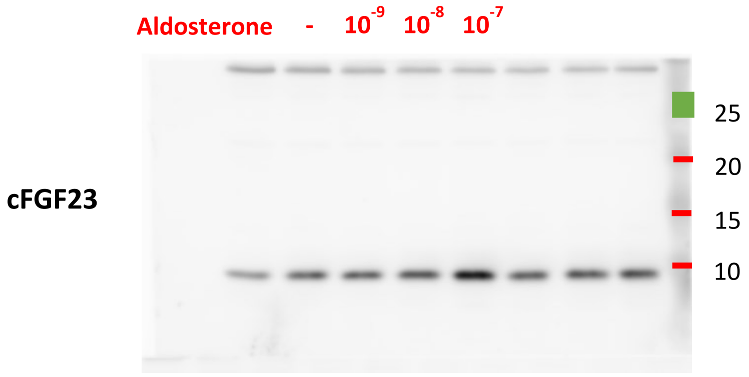

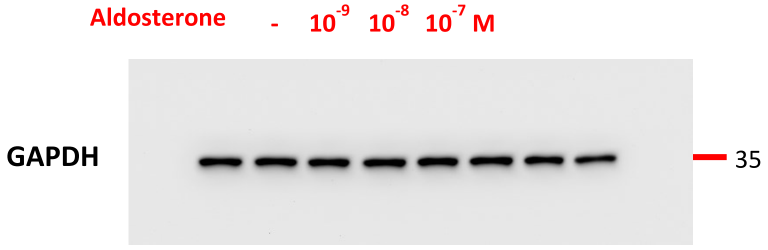

Figure 3B

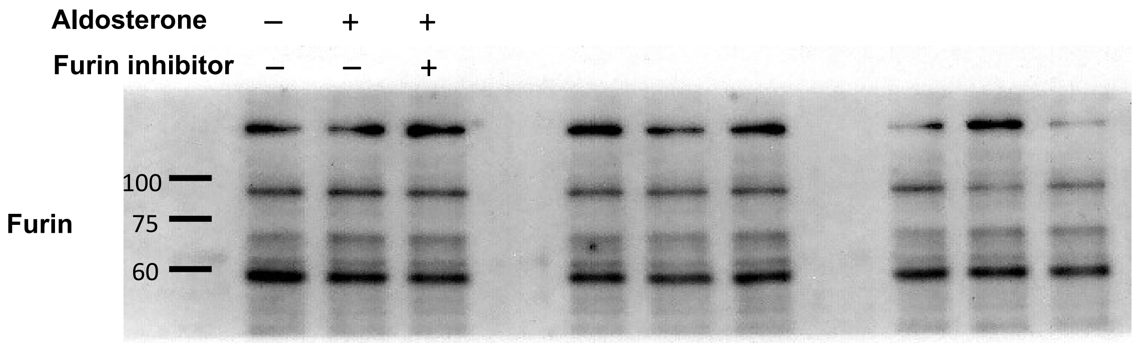

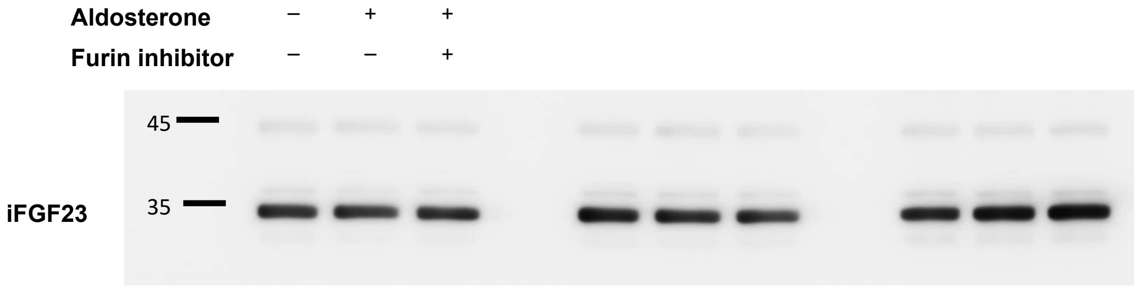

Figure 3B

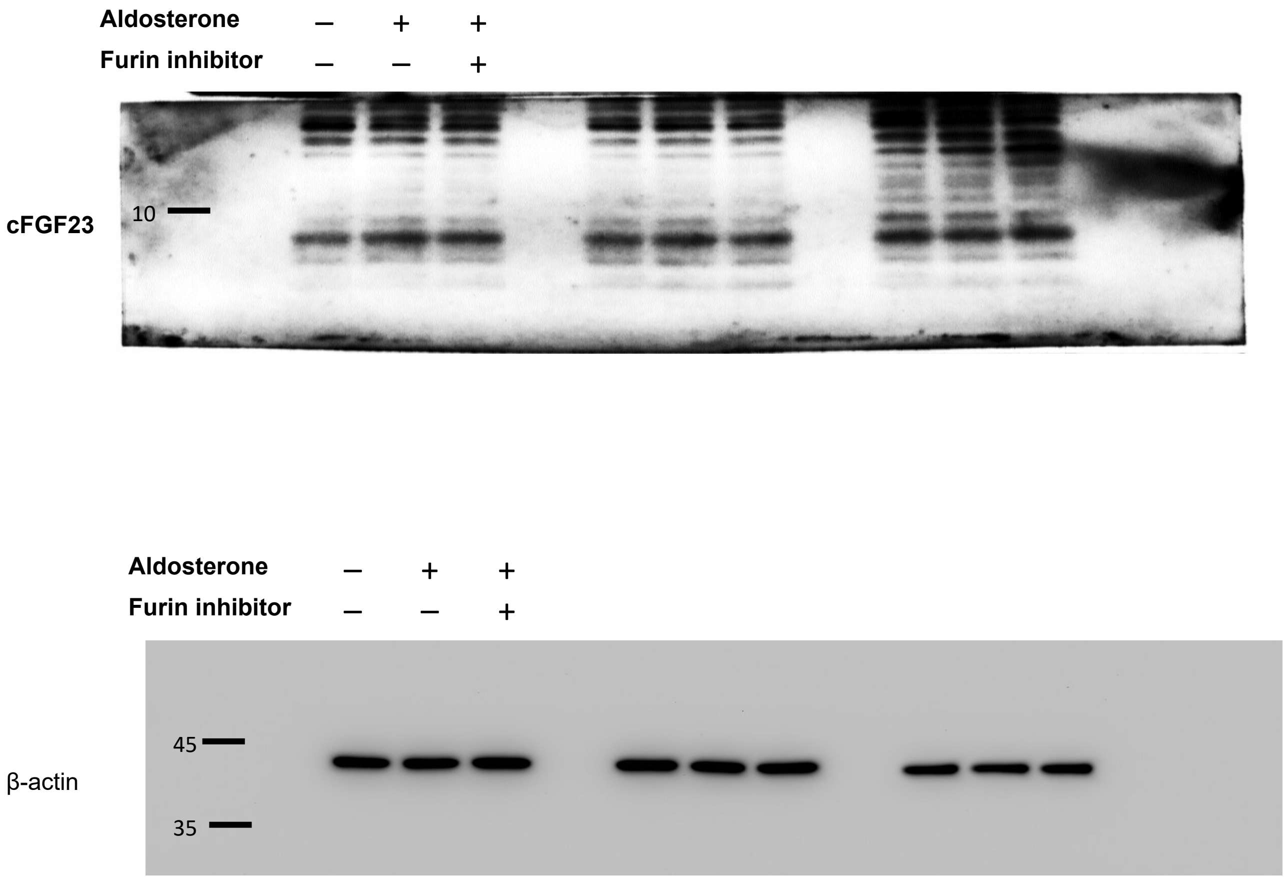

Figure 3C

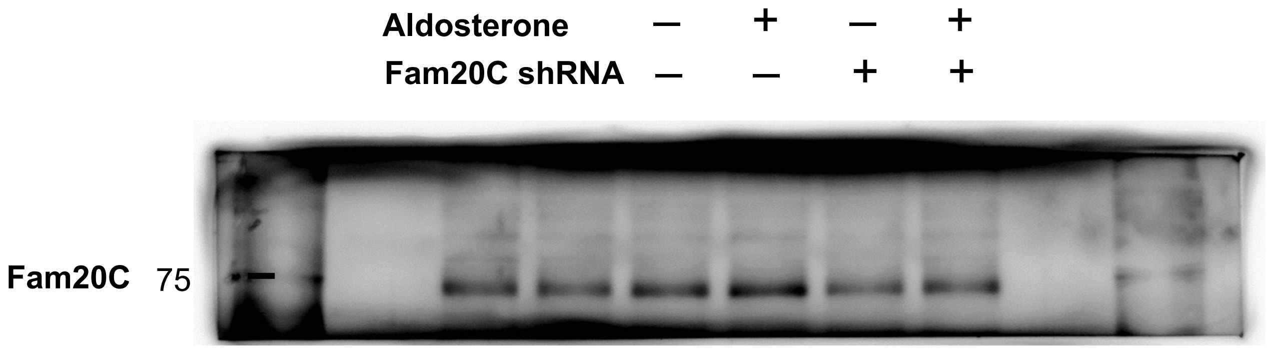

Figure 3C

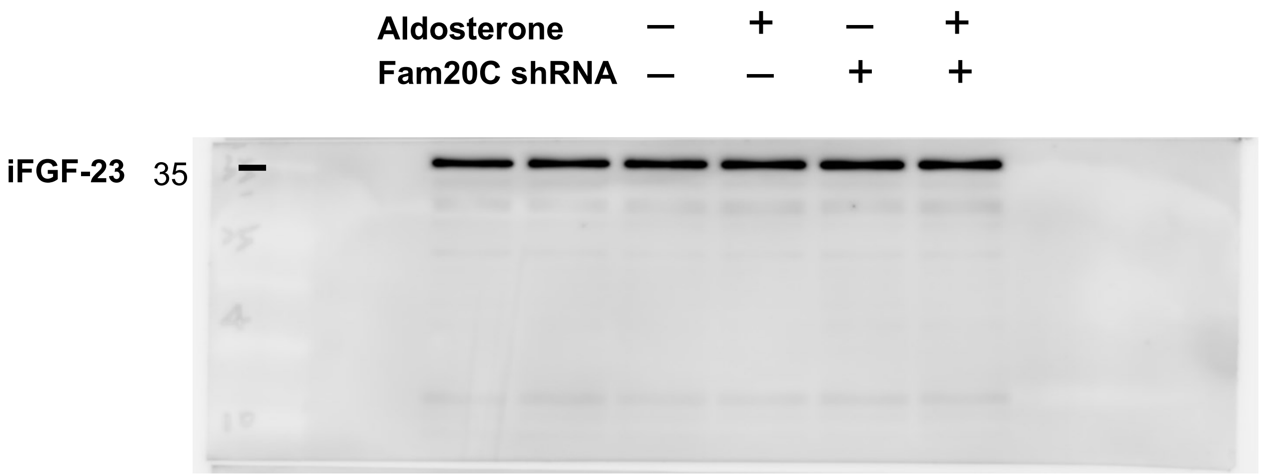
Aldosterone – + – +
Fam20C shRNA – – + +
iFGF-23
35

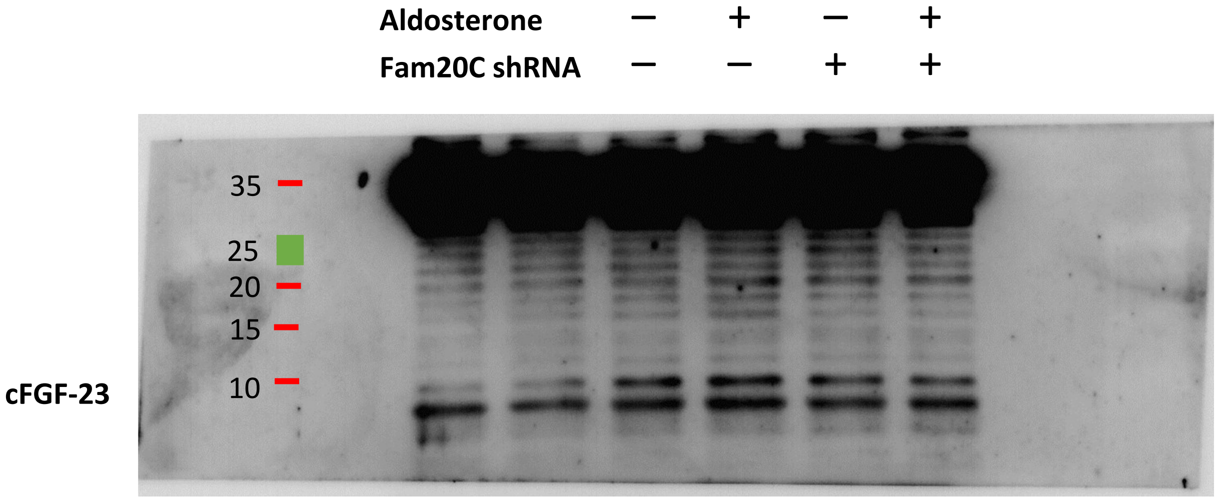
Aldosterone – + – +
Fam20C shRNA – – + +
35
25
20
15
10
cFGF-23

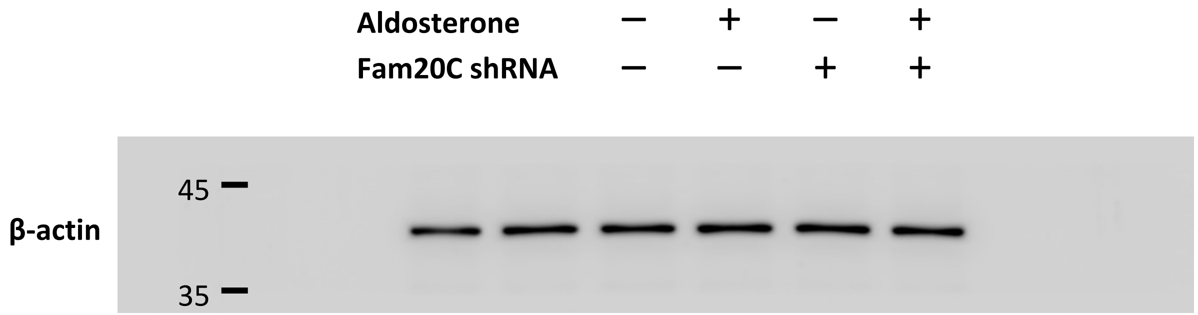
Aldosterone – + – +
Fam20C shRNA – – + +
45
β-actin
35

Figure 4A

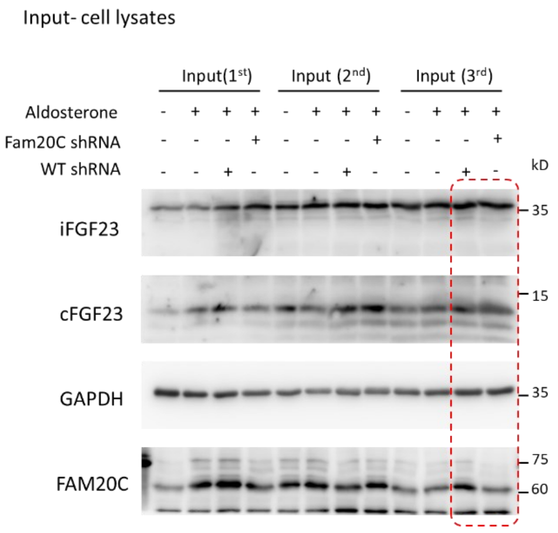

Figure 4B

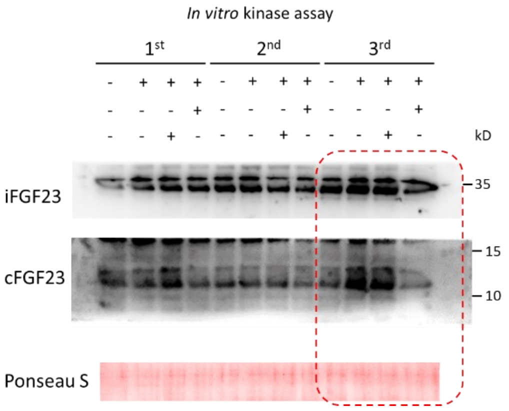

Supplementary Figure 7

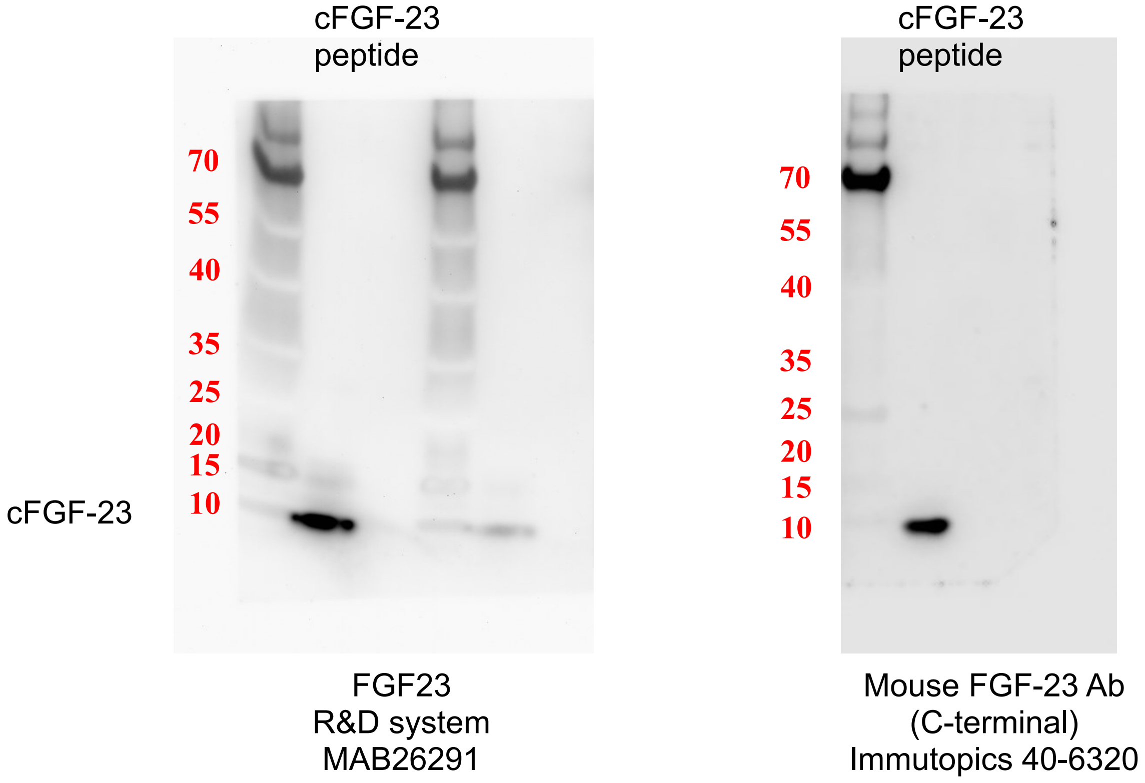

Supplement: Unedited blot and gel images [file jciinsight-10-166461-s066.pdf]
